# MicroRNA-519a-3p mediates apoptosis resistance in breast cancer cells and their escape from recognition by natural killer cells

**DOI:** 10.1038/cddis.2017.364

**Published:** 2017-08-03

**Authors:** Christian Breunig, Jens Pahl, Moritz Küblbeck, Matthias Miller, Daniela Antonelli, Nese Erdem, Cornelia Wirth, Rainer Will, Alexander Bott, Adelheid Cerwenka, Stefan Wiemann

**Affiliations:** 1 Division of Molecular Genome Analysis, German Cancer Research Center (DKFZ), Heidelberg, Germany; 2 Innate Immunity Group, German Cancer Research Center (DKFZ), Heidelberg, Germany; 3 Genomics & Proteomics Core Facilities, German Cancer Research Center (DKFZ), Heidelberg, Germany

## Abstract

Aggressive breast cancer is associated with poor patient outcome and characterized by the development of tumor cell variants that are able to escape from control of the immune system or are resistant to targeted therapies. The complex molecular mechanisms leading to immune escape and therapy resistance are incompletely understood. We have previously shown that high miR-519a-3p levels are associated with poor survival in breast cancer. Here, we demonstrate that miR-519a-3p confers resistance to apoptosis induced by TRAIL, FasL and granzyme B/perforin by interfering with apoptosis signaling in breast cancer cells. MiR-519a-3p diminished the expression of its direct target genes for TRAIL-R2 (*TNFRSF10B*) and for caspase-8 (*CASP8*) and its indirect target gene for caspase-7 (*CASP7*), resulting in reduced sensitivity and tumor cell apoptosis in response to apoptotic stimuli. Furthermore, miR-519a-3p impaired tumor cell killing by natural killer (NK) cells via downregulation of the NKG2D ligands ULBP2 and MICA on the surface of tumor cells that are crucial for the recognition of these tumor cells by NK cells. We determined that miR-519a-3p was overexpressed in more aggressive mutant *TP53* breast cancer that was associated with poor survival. Furthermore, low levels of TRAIL-R2, caspase-7 and caspase-8 correlated with poor survival, suggesting that the inhibitory effect of miR-519a-3p on TRAIL-R2 and caspases may have direct clinical relevance in lowering patient’s prognosis. In conclusion, we demonstrate that miR-519a-3p is a critical factor in mediating resistance toward cancer cell apoptosis and impairing tumor cell recognition by NK cells. This joint regulation of apoptosis and immune cell recognition through miR-519a-3p supports the hypothesis that miRNAs are key regulators of cancer cell fate, facilitating cancer progression and evasion from immunosurveillance at multiple and interconnected levels.

Breast cancer is the most common type of cancer in women worldwide and represents the second leading cause of cancer mortality in women.^1, 2^ The broad molecular and pathological heterogeneity of breast cancer subtypes is reflected by the diversity of the underlying biology and a particularly poor prognosis of some subtypes.^3, 4^ Targeted therapies have fundamentally improved patient outcome for estrogen receptor-positive luminal A as well as for HER2-positive breast cancer subtypes.^5^ However, particularly the luminal B and triple-negative breast cancer (TNBC) subtypes have remained clinical challenges.^6^ New treatment strategies for TNBC employ targeted therapies inhibiting PARP, PI3K or MEK in combination with apoptotic ligands such as TRAIL and with chemotherapy.^7, 8, 9, 10^ Chemotherapy as well as targeted therapies aim to reduce cell growth, survival as well as metastasis and/or induce apoptosis in breast cancer cells. However, their efficacy is limited by the development of therapy resistance and subsequent tumor progression.^11, 12, 13^ The molecular mechanisms leading to therapy resistance are diverse, often affect apoptosis at different levels in the signaling cascades involved and have remained incompletely understood.^14^

TRAIL efficiently induced apoptosis in several cell line models;^15^ however, it has been reported to even increase cell growth and metastasis formation in TRAIL-resistant tumors.^16, 17^ In addition to targeting the cancer cells directly, the (re-)activation of the immune system has become a promising strategy in current clinical trials for the treatment of solid tumors, including breast cancer.^18, 19^ Activated immune cells, like T and natural killer (NK) cells, can eliminate tumor cells by inducing apoptosis. Mechanistically, this is mediated via exocytosis of cytotoxic granules from NK cells, containing perforin and granzymes, as well as via induction of the TNF superfamily (Fas ligand and TRAIL) signaling pathways in the tumor cells.^20^ However, some tumors escape T-cell as well as NK-cell recognition and/or their killing machinery using mechanisms that are incompletely understood.^21, 22^

MicroRNAs (miRNAs) have previously been discovered to play pivotal roles in many biological processes including breast cancer development and regulation of apoptosis.^23, 24^ MiRNAs are small non-protein-coding RNAs of ∼22 nucleotides and are mostly negative regulators of gene expression by targeting the three-prime untranslated regions (3′UTRs) of target messenger RNAs (mRNAs). Recent progress in cancer biology has revealed that miRNAs are frequently deregulated in various human cancers, including breast cancer, thereby promoting cancer development and induction of drug resistance.^25, 26, 27^

We have previously identified miRNA 519a-3p (miR-519a-3p) to be upregulated in tamoxifen-resistant breast cancer cells.^28, 29^ MiR-519a-3p targets several tumor suppressor genes, thereby increasing cell viability and cell cycle progression.^29^ In the present study, we analyzed the effects that miR-519a-3p has in TRAIL and Fas ligand (FasL-)-mediated induction of apoptosis. We specifically investigated the effects of miR-519a-3p on the susceptibility of breast cancer cells toward NK cell-mediated cytotoxicity as a potential mechanism for tumor cell escape from immune cell recognition and a rather physiological trigger for apoptosis. We show that miR-519a-3p indeed leads to inhibition of TRAIL- and FasL-induced apoptosis in breast cancer cell lines by directly targeting the proapoptotic *TNFRSF10B* (TRAIL-R2) and *CASP8* (caspase-8) mRNAs. Moreover, miR-519a-3p decreases NK cell-mediated killing of breast cancer cells by downregulating tumor cell ligands for the NK cell-activating receptor NKG2D and conferring resistance toward granzyme B- as well as TRAIL-induced apoptosis. Consequently, we propose a model in which miR-519a-3p is involved in aggressive and/or therapy-resistant breast cancer by facilitating evasion of NK cell recognition as well as by inducing resistance toward apoptosis formation.

## Results

### TRAIL-R2, caspase-7 and caspase-8 are regulated by miR-519a-3p

In a previous study, we had identified miR-519a-3p as an oncomiR in luminal A breast cancer that is upregulated in tamoxifen-resistant MCF-7 breast cancer cells and increases cell viability as well as cell cycle progression.^28, 29^ Here, we set out to investigate the impact that miR-519a-3p has within breast cancer cells in response toward apoptotic stimuli. Using TargetScan (version 7.1, http://www.targetscan.org/vert_71/) and DAVID (version 6.7, https://david.ncifcrf.gov/) we identified enriched KEGG pathways predicted to be targeted by miR-519a-3p. The apoptosis signaling pathway (e.g., through death ligands FasL and TRAIL) was among the top (Supplementary Table S1 and Supplementary Figure S1).

**Figure 1:**
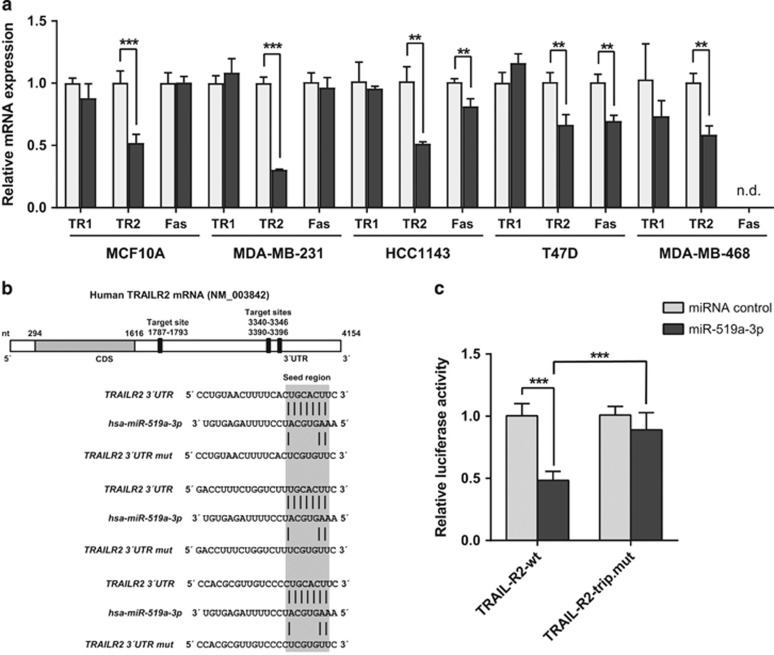
TRAIL-R2 is a direct target of miR-519a-3p. (**a**) qRT-PCR reveals downregulation of TRAIL-R2 (TR2), but not TRAIL-R1 (TR1) and Fas in MCF10A, MDA-MB-231, HCC1143, T47D and MDA-MB-468 cells. Cells were transfected with miRNA control or miR-519a-3p for 48 h, mRNA was isolated and gene expression of TRAIL-R1, TRAIL-R2 and Fas was analyzed (*n*=3). (**b**) Schematic representation of the miR-519a-3p target sites within the 3′UTR of *TNFRSF10B* (TRAIL-R2) mRNA. (**c**) Luciferase reporter assays were performed using psiCHECK constructs in MCF-7 cells. Luciferase activity of *TNFRSF10B* 3′UTR is reduced after miR-519a-3p transfection compared with control. The signal of the wt UTR (TRAIL-R2-wt) is rescued after mutating all three binding sites for miRNA-519a-3p (TRAIL-R2-trip.mut) (*n*=6). Data are expressed as mean+S.D.; **P<0.01, ***P<0.001. All *P-*values are based on analysis of miRNA control *versus* miR-519a-3p

In accordance with these *in silico* data, overexpression of miR-519a-3p consistently reduced the expression of *TNFRSF10B* (TRAIL-R2) but not of *TNFRSF10A* (TRAIL-R1) or *FAS* mRNA in the immortalized but nontransformed MCF10A epithelial cell line as well as in several breast cancer cell lines, MDA-MB-231, HCC1143, T47D and MDA-MB-468 (Figure 1a). In addition, cell surface expression of TRAIL-R2 was reduced in these conditions, indicating that miR-519a-3p also negatively regulated TRAIL-R2 expression at the protein level (Supplementary Figure S2).

To determine whether reduced expression of TRAIL-R2 resulted from direct targeting of miR-519a-3p to the 3′UTR of *TNFRSF10B* mRNA, a reporter gene construct was cloned with the 3′UTR of the *TNFRSF10B* gene downstream of the *Renilla* luciferase open reading frame (Figure 1b). Co-transfection of this reporter gene construct with miR-519a-3p revealed that miR-519a-3p specifically reduced relative luciferase reporter activity (Figure 1c). Next, we introduced mutations into all three predicted miR-519a-3p binding sites within the reporter gene construct by mutating four nucleotides each within the seed-matching sequences of the UTR to validate specificity of miR-519a-3p targeting (Figure 1b). Disruption of all three putative target sites indeed abrogated the miR-519a-3p-mediated reduction in luciferase activity and confirmed that TRAIL-R2 is a direct target of miR-519a-3p (Figure 1c).

**Figure 2:**
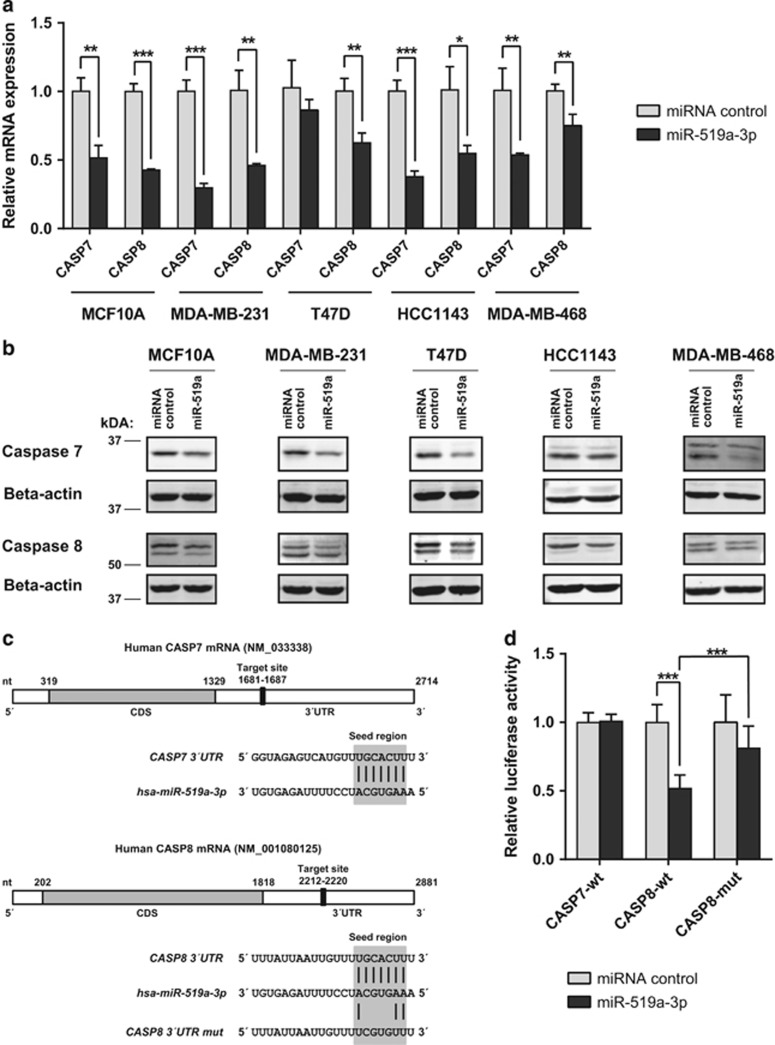
MiR-519a-3p downregulates caspases-7 and -8 at RNA and protein levels. (**a**) qRT-PCR reveals downregulation of *CASP7* and *CASP8* by miR-519a-3p in MCF10A, MDA-MB-231, T47D, HCC1143 and MDA-MB-468 cells (*n*=3). (**b**) Western blot analysis confers qRT-PCR results showing downregulation of caspases-7 and -8 by miR-519a-3p (miR-519a). *β*-Actin was used as a loading control. (**c**) Schematic representation of the miR-519a-3p target sites within the 3′UTR of caspase-7 and caspase-8 mRNA. (**d**) Luciferase activity is reduced after miR-519a-3p transfection compared with control with the wild-type *CASP8* (CASP8-wt) but not with the *CASP7* (CASP7-wt) 3′UTR in MCF10A cells. The signal is rescued after mutating binding site for miRNA-519a-3p (CASP8-mut) (*n*=6). Data are expressed as mean+S.D.; *P<0.05, **P<0.01, ***P<0.001. All *P-*values are based on analysis of miRNA control *versus* miR-519a-3p

In addition to TRAIL-R2, caspases-7 and -8, both key players in apoptosis induction by, for example, TRAIL and FasL,^30^ were predicted to be targeted by miR-519a-3p (Supplementary Figure S1). In line with the predictions, overexpression of miR-519a-3p reduced mRNA as well as protein expression of both caspases in MCF10A cells as well as in MDA-MB-231, HCC1143, T47D and MDA-MB-468 breast cancer cell lines (Figures 2a and b). Again employing luciferase reporter assays and site-directed mutagenesis, we identified *CASP8* as another direct target of miR-519a-3p (Figures 2c and d). In contrast, miR-519a-3p did not alter the luciferase activity when the 3′UTR reporter gene construct for *CASP7* was tested (Figure 2d).

In conclusion, we demonstrated that miR-519a-3p is a direct regulator of TRAIL-R2 and caspase-8 expression, whereas the modulation of caspase-7 expression appears to be indirect.

### MiR-519a-3p inhibits apoptosis induction by TRAIL and Fas ligand via TRAIL-R2, caspase-7 and caspase-8

**Figure 3:**
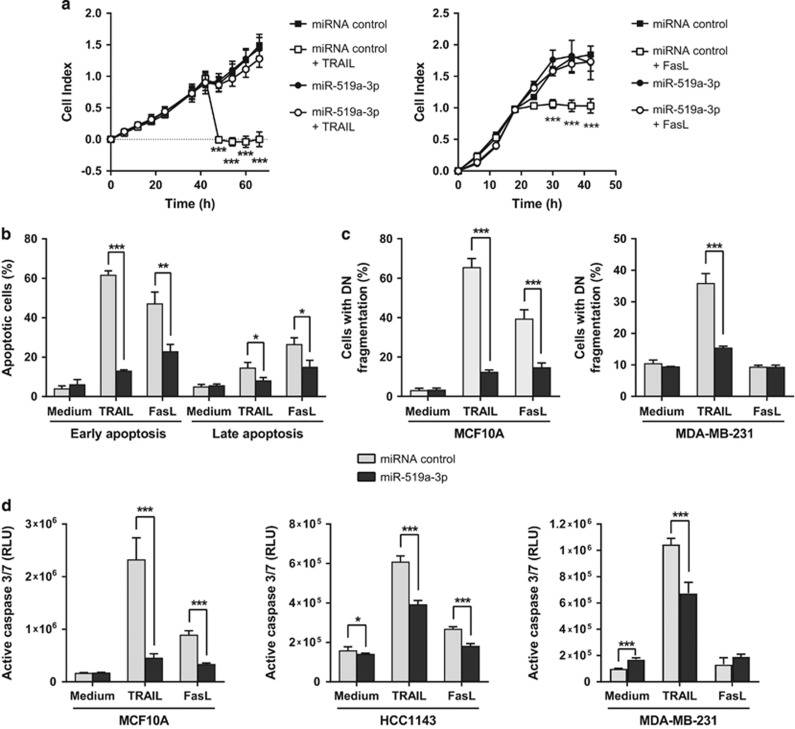
Elevated expression of miR-519a-3p induces resistance toward apoptosis induction. (**a**) RTCA (real-time cell analyzer) viability assay of MCF10A cells transfected with microRNA control or miR-519a-3p. Transfected cells were seeded in E-16 plates and were then treated with TRAIL (at *t*=36 h) or FasL (*t*=18 h). TRAIL (250 ng/ml; left panel) and FasL (10 *μ*g/ml; right panel) treatment induced a decrease in cell index (as proxy for cell viability) in miRNA control-transfected but not miR-519a-3p-transfected MCF10A cells (*n*=6). (**b**) TRAIL- and FasL-induced apoptosis was reduced upon overexpression of miR-519a-3p in MCF10A cells using Annexin V and 7-AAD. MCF10A were transfected with miR-519a-3p or miRNA control for 48 h and then treated with 60 ng/ml TRAIL, 5 *μ*g/ml FasL or medium control for additional 24 h. Shown is the analysis of early (Annexin V positive and 7-AAD negative) and late (Annexin V positive and 7-AAD positive) apoptosis (*n*=3). (**c**) TRAIL (60 ng/ml)-induced DNA fragmentation was reduced by miR-519a-3p in MCF10A and MDA-MB-231 cells, whereas FasL (5 *μ*g/ml)-induced DNA fragmentation was reduced by miR-519a-3p only in MCF10A. (**d**) Activation of caspase-3/7 activity in MCF10A, HCC1143 and MDA-MB-231 cells by TRAIL and FasL (except in MDA-MB-231) was strongly reduced by miR-519a-3p (*n*=8). Data are expressed as mean±S.D.; *P<0.05, **P<0.01, ***P<0.001. All *P-*values are based on analysis of miRNA control *versus* miR-519a-3p

We next investigated whether increased levels of miR-519a-3p would potentially protect from TRAIL- and FasL-induced apoptosis, as we had observed that the induction of apoptosis by TRAIL and FasL was mediated by TRAIL-R2 and caspase activation in some cell lines (Supplementary Figures S3 and S4). To this end, MCF10A as well as several breast cancer cell lines were transfected with miR-519a-3p or a control miRNA and were then treated with TRAIL or anti-Fas antibody (FasL) to induce apoptosis. Indeed, only MCF10A cells overexpressing miR-519a-3p retained normal cell viability despite TRAIL or anti-Fas treatment (Figure 3a). To substantiate that this effect was due to inhibition of apoptosis, MCF10A, MDA-MB-231, HCC1143 and T47D cells were incubated with TRAIL or FasL, and were then tested for the activities of extrinsic and intrinsic apoptosis signaling pathways.^31^ Indeed, early (Annexin V positive and 7-AAD negative) and late (Annexin V positive and 7-AAD positive) apoptosis (Figure 3b and Supplementary Figure S5a), DNA fragmentation (Figure 3c), caspase-3/7 activity (Figure 3d and Supplementary Figure S5B) and loss of mitochondrial potential (Supplementary Figure S5c) were all specifically impaired in miR-519a-3p-overexpressing cells. Similarly, stable transfection of MDA-MB-231 cells with miR-519a-3p induced resistance toward TRAIL treatment (Supplementary Figures S5d and e). Reducing miR-519a-3p levels with antagomirs in MCF10A cells as well as in MDA-MB-231 stably expressing miR-519a-3p led to an increase in apoptosis induction upon TRAIL treatment (Supplementary Figures S6a and b). Of note, MDA-MB-231 cells were resistant toward FasL-induced apoptosis (Figures 3c and d as well as Supplementary Figures S3a and S5a and c) even though they express Fas at low levels at the cell surface (Supplementary Figure S2).

We next wanted to know whether miR-519a-3p overexpression would also confer resistance to other apoptosis-inducing agents. Indeed, miR-519a-3p protected cells from apoptosis upon treatment with staurosporine, a potent inducer of apoptosis through both caspase-dependent and caspase-independent mechanisms (Supplementary Figure S7a).^32^ To test whether miR-519a-3p can influence the effect of chemotherapeutic drugs typically used in the treatment of patients with aggressive breast cancer, miR-519a-3p-transfected MDA-MB-231 breast cancer cells were treated with paclitaxel (Taxol). Indeed, viability was reduced in paclitaxel-treated cells, in a dose-dependent manner, and to a lesser extent in miR-519a-3p-overexpressing cells as compared with control cells. These results suggest that miR-519a-3p can protect tumor cells from chemotherapy, like paclitaxel (Supplementary Figure S7b).

These data support a mechanistic model in which miR-519a mediates resistance to TRAIL and Fas ligand as well as to chemotherapeutic drugs via blockade of apoptosis.

### MiR-519a-3p inhibits NK cell-mediated cytotoxicity by reducing the surface expression of NKG2D ligands on tumor cells

**Figure 4:**
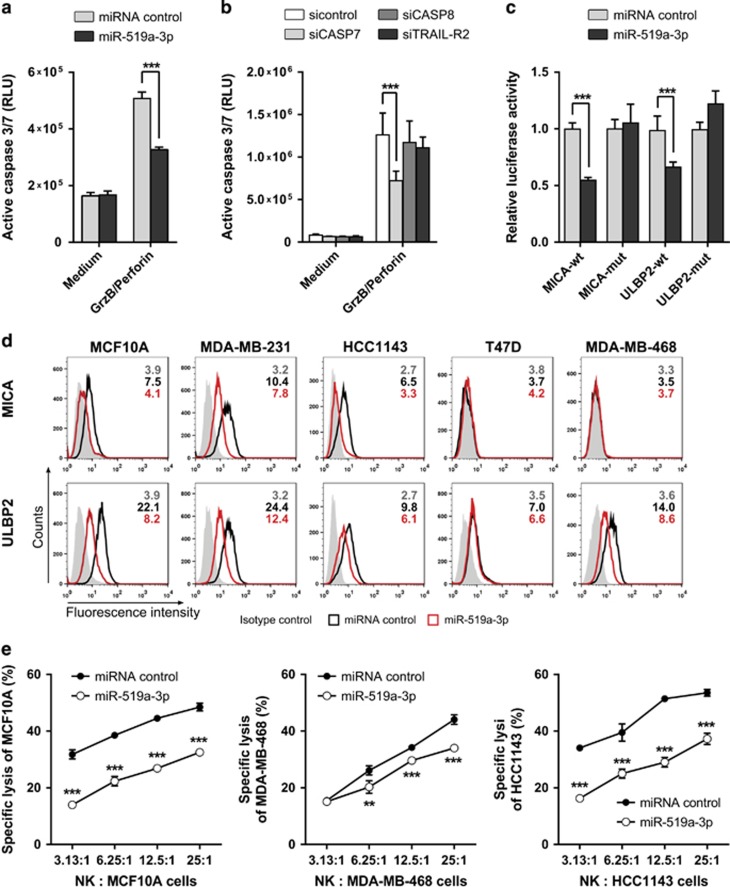
MiR-519a-3p inhibits NK cell-mediated cytotoxicity via caspase-7, MICA and ULBP2. (**a**) MCF10A cells were treated with granzyme B (GrzB) and perforin for 6 h and caspase-3/7 activity was measured. MiR-519a-3p reduced granzyme B/perforin-induced apoptosis induction (*n*=5). (**b**) Granzyme B/perforin-induced apoptosis was reduced by silencing of *CASP7* but not of *CASP8* in MCF10A cells. Cells were transfected with siTRAIL-R2, siCASP7, siCASP8 and miRNA control for 48 h and then treated with granzyme B/perforin for 6 h and active caspase-3/7 was measured (*n*=8). (**c**) Luciferase reporter assays were performed using psiCHECK constructs in MCF-7 cells. Luciferase activity was reduced in the presence of MICA and ULBP2 3′UTRs after miR-519a-3p transfection compared with miRNA control. The signal was rescued after mutating the respective binding sites for miRNA-519a-3p (*n*=5). (**d**) FACS analysis results showing downregulation of MICA and ULBP2 in breast cancer cells. Cells were transfected with miRNA control or miR-519a-3p for 48 h and cells were stained for MICA and ULBP2. Median fluorescence intensity for isotype control (gray), miRNA control (black) and miR-519a-3p (red) is depicted in each histogram. Shown is one representative FACS plot of at least three experimental repeats. (**e**) ^51^Cr release assay of MCF10A, MDA-MB-468 and HCC1143 cells after transfection with miR-519a-3p or miRNA control and subsequent co-culture with primary human NK cells in indicated ratios. Lysis of MCF10A, MDA-MB-468 and HCC1143 was measured in a 4 h ^51^Cr release assay (*n*=3). Data are expressed as mean+S.D.; **P<0.01, ***P<0.001. All *P-*values are based on analysis of miRNA control *versus* miR-519a-3p or siRNA control *versus* siCASP7, siCASP8 and siTRAILR2

Killing of cancer cells by NK cells is also mediated by the cytolytic proteins perforin, granzymes as well as TRAIL, all inducing apoptosis in target cells.^33, 34, 35^ Of note, we found granzyme B-induced apoptosis and caspase-7 activation to be reduced by miR-519a-3p (Figures 4a and b). Hence, to test in a more physiological context whether miR-519a-3p mediates apoptosis resistance, we investigated whether miR-519a-3p might protect breast cancer cells from killing by human primary NK cells. We identified MICA and ULBP2, two key ligands for the NK cell-activating receptor NKG2D, among the predicted miR-519a-3p target genes (Supplementary Figure S8). Killing of MCF10A, MDA-MB-468 and HCC1143 cells by NK cells was indeed reduced after blockage of NKG2D on NK cells using an anti-NKG2D antibody or upon siRNA-mediated knockdown of MICA or ULBP2 in tumor cells. This shows that the NKG2D ligands MICA and ULBP2 are crucial for NK cell cytotoxicity against these breast cancer cells (Supplementary Figure S9).

To corroborate the targeting of MICA and ULPB2 by miR-519a-3p, we identified potential miR-519a-3p binding sites in the 3′UTR of the genes of MICA and ULBP2 using TargetScan (Supplementary Figure S10). Luciferase reporter assays and using wild-type and mutated 3′UTR binding sites of miR-519a-3p revealed that MICA and ULBP2 were both direct targets of miR-519a-3p (Figure 4c). Indeed, miR-519a-3p reduced the mRNA levels and surface protein levels of MICA and ULBP2 in MCF10A, MDA-MB-231, HCC1143, T47D as well as MDA-MB-468 cells, whereas other NKG2D or DNAM-1 ligands like ULBP1, ULBP3, ULBP4, MICB and CD155 were not significantly affected by miR-519a-3p (Figure 4d and Supplementary Figures S11a and b).

To test whether resistance to apoptosis as well as downregulation of NK cell ligands by miR-519a-3p results in lower NK cell-mediated lysis of breast cancer cells, we co-cultured ^51^Cr-loaded MCF10A, MDA-MB-468 and HCC1143 cells with IL-2-activated NK cells and then analyzed ^51^Cr release as a measure for tumor cell killing by NK cells. When overexpressed with miR-519a-3p, the lysis of tumor cells by NK cells was significantly reduced as compared with tumor cells transfected with a control miRNA (Figure 4e). This reduced lysis of miR-519a-3p-overexpressing cells was restored in the presence of a miR-519a-3p antagomir (Supplementary Figure S12), confirming specificity of the miRNA effect. In addition, although NK cell degranulation, based on externalization of CD107a, was abrogated toward miR-519a-3p-overexpressing MCF10A cells, NK cell degranulation was rescued when miR-519a-3p-overexpressing MCF10A cells additionally overexpressed MICA and ULBP2 (Supplementary Figure S13). Thus, miR-519a-3p impaired NK cell activation and degranulation by the downregulation of MICA and ULPB2.

Collectively, these data demonstrate that miR-519a-3p impairs recognition and killing of breast cancer cell by NK cells via direct targeting of *MICA* and *ULBP2* in addition to inhibiting caspase-7-induced apoptosis.

### Low levels of caspases-7 and -8 as well as TRAIL-R2 correlate with poor disease-free survival and miR-519a-3p is higher expressed in advanced-grade breast cancer

**Figure 5:**
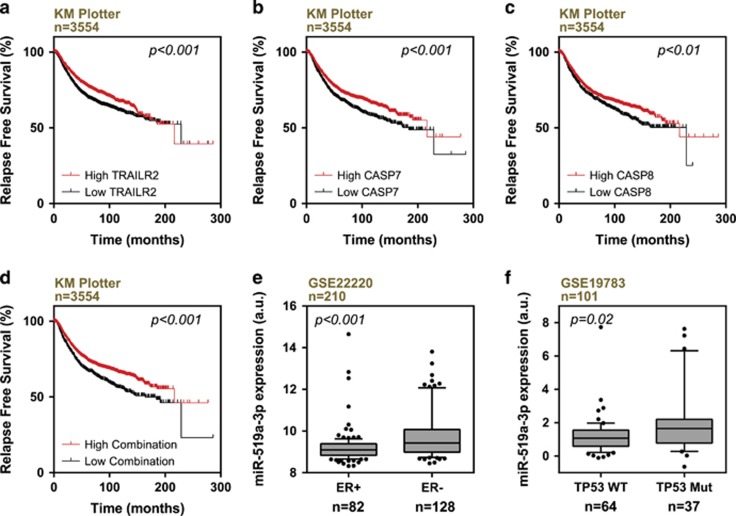
Correlation of *CASP7/8* and *TNFRSF10B* levels with survival outcome and miR-519a-3p with mutant *TP53* expression. (**a–c**) Low expression of *TNFRSF10B*, *CASP7* and *CASP8* (black curves) correlate with poorer survival of breast cancer patients (KM Plotter). (**d**) Lower combined mean expression of *TNFRSF10B*, *CASP7* and *CASP8* expression (black curve) correlate with poorer survival of breast cancer patients (KM Plotter). (**e**) Analysis of breast cancer patient data set GSE22220 revealed that miR-519a-3p is higher expressed in estrogen receptor-negative (ER−) compared with estrogen receptor-positive (ER+) tumors. (**f**) Analysis of breast cancer patient data set GSE19783 revealed that miR-519a-3p is higher expressed in tumors with mutated in *TP53* (TP53 Mut) than in tumors with wild-type *TP53* (TP53 WT)

We and others have shown clinical relevance of miR-519a-3p and MICA/ULBP2 in breast cancer.^29, 36^ Although low levels of *MICA* and *ULBP2* correlate with poor clinical outcome in breast cancer patients,^36^ miR-519a-3p is elevated in higher-grade breast cancer^29^ and correlates with reduced relapse-free survival (Supplementary Figures S14a and b). Here, we analyzed the impact of the expression of direct and indirect miR-519a-3p target genes *CASP8*, *TNFRSF10B* and *CASP-7* on the clinical outcome using published breast cancer data sets. Low expression of *CASP7*, *CASP8* and *TNFRSF10B*, alone and in combination, correlated with shorter relapse-free survival (Figures 5a–d and Supplementary Figure S14C). These findings are in line with our experimental data and suggest a causal role of miR-519a-3p in aggressive breast cancer.

This is further supported by our observation that miR-519a-3p was higher expressed in estrogen receptor-negative (ER−) than in estrogen receptor-positive (ER+) breast cancer (Figure 5e) as well as in ER− breast cancer cell lines.^29^ The ER− breast cancer is mostly associated with mutations in *TP53* and associated with poor survival.^37^ Clinical data show that miR-519a-3p expression was elevated in breast tumors with mutated *TP53* (Figure 5f) and that the latter was correlated with poor relapse-free survival (Supplementary Figure S15).

Altogether, our findings reveal that high levels of miR-519a-3p as well as low levels of *CASP7*, *CASP8* and *TNFRSF10B* correlate with poor disease-free survival.

## Discussion

**Figure 6:**
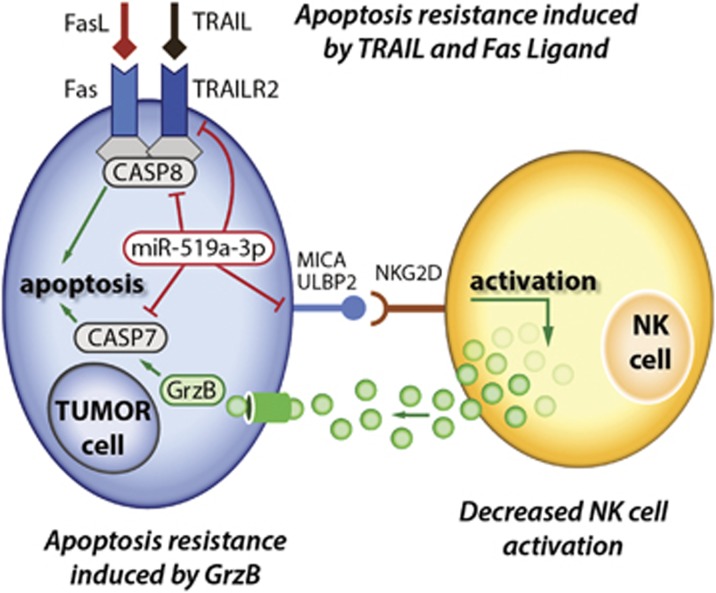
Mechanistic model of miR-519a-3p activities negatively regulating apoptosis induction and NK cell activation. Higher expression of miR-519a-3p blocks TRAIL- as well as FasL-induced apoptosis signaling pathways by decreasing TRAIL-R2, caspase-7 and caspase-8 expression. Furthermore, the miRNA blocks granzyme B/perforin-induced apoptosis by decreasing caspase-7. Finally, miR-519a-3p abrogates NK cell activation through reduced expression of MICA and ULBP2. Hence, miR-519a-3p induces escape of breast cancer cells from NK cell-mediated cytotoxicity and resistance toward apoptosis induction, thus synergistically contributing to cancer cell survival

Breast cancer is a highly heterogeneous disease and the clinical outcome strongly correlates with the respective tumor subtype.^1^ Deregulated apoptosis, mostly via mutations in *TP53*, is a common driving factor in most tumor diseases.^38^ Hence, the induction of apoptosis in tumor cells is a highly relevant mechanism in anticancer therapy.^14^ Currently, there are several strategies for targeting apoptosis in breast cancer immunotherapy and chemotherapy. Besides TRAIL and FasL, apoptosis can be induced by various stimuli and through diverse mechanisms.^32, 39, 40^ However, development of resistance toward apoptosis is one major clinical challenge.^14, 21^ Anthracyclines and taxanes have remained major first-line chemotherapies in treatment of metastatic luminal B and TNBC^11^ despite the development of acquired or *de novo* resistance in metastatic and high-grade breast cancer.^41^ Ajabnoor *et al.*^42^ showed that paclitaxel resistance is associated with diminished apoptotic response by loss of caspase-mediated cell death. In the present study, our data indicate that miR-519a-3p contributes to resistance to TRAIL, FasL, granzyme B/perforin as well as to paclitaxel treatment. In addition, miR-519a-3p reduces activation and cytotoxicity of NK cells, suggesting that this miRNA could play an important role in the regulation of apoptosis in breast cancer. Our findings are corroborated by breast cancer patient data, showing that high expression of miRNA-519a-3p and low coexpression of the target genes *CASP7*, *CASP8* and *TNFRSF10B* correlate with poor relapse-free survival in breast cancer patients. Consequently, we show here for the first time that miR-519a-3p is involved in resisting cell death and avoiding immune destruction of breast cancer cells at the same time. On the one hand, miR-519a-3p influences the tumor microenvironment by facilitating evasion of NK cell recognition and, on the other hand, induces resistance toward apoptosis formation within the breast cancer cells, as schematically summarized in Figure 6.

In recent years, genomic and transcriptomic data of breast cancer samples have led to a subclassification of breast cancer subtypes.^3, 43^ The correlation with breast cancer subtypes as well as with poor or good prognosis of patients has been demonstrated for several miRNAs.^27, 44, 45, 46^ MiRNAs have been described as regulators of many different biological processes, including immune and apoptosis regulation in cancer, and thereby shaping the tumor microenvironment.^24, 47, 48, 49, 50, 51^ We and others have previously described that miR-519a-3p promotes proliferation in tamoxifen-resistant ER+ breast cancer and hepatocellular carcinoma.^29, 52, 53^ Using publically available data sets, we observed that higher miR-519a-3p expression associates with poor survival of breast cancer patients.^29^ Elevated miR-519a-3p levels were found particularly in more aggressive ER− as well as in histopathologic grade 3 breast cancer subtypes. TNBC often harbors *TP53* mutations and, using publicly available data sets, we found that specifically *TP53* mutant breast tumors express elevated levels of miR-519a-3p. *TP53* mutations are rare in small and low-grade tumors, whereas the frequency increases with the size and grade of tumors.^54^ Furthermore, breast cancer patients with somatic *TP53* mutations have shorter disease-free survival compared with patients with wild-type *TP53*, and respond less well to chemotherapy, antihormonal therapy or radiotherapy.^55, 56^ In future studies it has to be analyzed whether elevation of miR-519a-3p in *TP53-*mutated patients is directly regulated by p53 and/or other factors.

NK cell-mediated clearance of cancerous cells is a central intrinsic mechanism counteracting tumor development. Activated NK cells can induce tumor cell apoptosis via membrane-bound or soluble TRAIL and Fas ligands as well as by releasing granules that contain perforin and granzymes.^33, 34^ Breast cancer cells have been described to evade immunosurveillance that may be imposed by tumor-intrinsic factors or the immunosuppressive cancer microenvironment.^22, 57, 58^ They can escape NK cell-mediated cytotoxicity by diverse mechanisms that are independent of the breast cancer subtype.^22, 59^ Besides this, breast cancer cells are often resistant against apoptosis induction, thus limiting the clinical effectiveness of apoptosis-inducing agents.^21, 47, 60^

In this study, we demonstrate that miR-519a-3p is a novel tumor-intrinsic factor, regulating breast cancer cell recognition and killing by NK cells. We identified miR-519a-3p to induce resistance toward NK cell-mediated cytotoxicity by inhibiting FasL-, TRAIL- and granzyme B-induced apoptosis. TRAIL and FasL bind to their corresponding receptors on target cells, thereby inducing apoptosis through the extrinsic and intrinsic apoptosis pathways. It has previously been revealed that several genes and miRNAs either directly or indirectly regulate the TRAIL-induced apoptosis pathway.^61^ Here, we have shown that miR-519a-3p confers resistance toward TRAIL- and FasL-induced apoptosis by downregulating both TRAIL-R2 and caspase-8. This is in accordance to Zhang and Zhang^60^ who reported that constitutively endocytosed TRAIL-R1 and TRAIL-R2 led to TRAIL resistance. Upon binding of TRAIL to its receptors TRAIL-R1 and TRAIL-R2, endogenous FADD and caspase-8 become recruited to the receptor.^62^ In the present work, we show that caspase-8 is required for apoptosis induction by TRAIL and FasL and that expression of *CASP8* is correlated with patient survival. Caspase-7 was also downregulated by miR-519a-3p, but it does not seem to be a direct target. MacFarlane *et al.*^63^ reported that caspase-7 gets activated upon stimulation with TRAIL in MCF-7 cells, whereas our data indicate that caspase-7 is not required for TRAIL- and FasL-induced apoptosis in MCF10A and MDA-MB-231 cell lines. However, caspase-7 is indeed necessary for TRAIL- and FasL-induced apoptosis in HCC1143 and T47D breast cancer cells, suggesting a cell line- or cell type-specific effect of caspase-7. In addition to TRAIL and FasL, NK cells kill target cells by releasing granules containing perforin and granzymes.^64^ Martin and colleagues^65, 66^ revealed that granzyme B can promote apoptosis by direct processing of effector caspases-3 and -7. This is consistent with our data that suggest a key function of caspase-7 in granzyme-B mediated apoptosis.

NK cell-mediated cytotoxicity is regulated by inhibitory killer cell immunoglobulin-like receptors (KIRs) and activating receptors including NKG2D expressed on NK cells.^59^ We and others have previously shown that MICA, MICB and ULBPs are important factors in the activation of NK cells.^67, 68, 69^ In tumor cells, different mechanisms enable their escape from recognition by NK cells, such as the secretion of soluble inhibitory factors or the downregulation of activating ligands.^59^ Previous studies have shown that miRNAs of the miR-17-92 cluster, miR-10b, and miR34a/c mediate the escape of tumor cell recognition by NK cells by targeting MICA, MICB and ULBP2, respectively.^49, 70, 71, 72^ Complementary, altered miR-183 expression in NK cells silenced their antitumor cytotoxic potential by targeting DAP12.^73^ In this study, we demonstrate that the NKG2D ligands MICA and ULBP2 are downregulated by miR-519a-3p in breast cancer cells MDA-MB-231, HCC1143, T47D, MDA-MB-468 as well as in MCF10A cells, thereby attenuating NK cell activation and reducing NK cell-mediated cytotoxicity. This effect was effectively abrogated by blocking miR-519a-3p with antagomirs. Suppression of NKG2D on primary human NK cells or downregulation of MICA or ULBP2 with siRNA mimicked the effect of miR-519a-3p and reduced NK cell activation as well as cytotoxicity that is in line with previous studies on NKG2D function.^59, 67, 68, 72^ In addition, low levels of MICA/B and ULBPs correlate with poor clinical outcome in breast cancer patients.^36^ Besides NK cells, NKG2D is also expressed on the cell surface of human CD8^+^ T cells and transmits a costimulatory signal.^74^ Future research will be required to unravel the effect of miR-519a-3p on CD8^+^ T-cell activation via NKG2D.

In conclusion, overcoming apoptosis resistance and tumor immune escape are key switches to successfully develop targeted apoptosis and immune therapies. Although miRNAs often do not act alone but rather in concert and on a range of mRNAs and genes in tumor cells^26^ and in stromal cell types,^48^ we here provide evidence that miR-519a-3p promotes cancer cell survival by a combination of abrogated NK cell activation as well as development of resistance toward NK cell-mediated cytotoxicity and induction of tumor cell apoptosis by synergistically regulating functionally connected pathways.

## Materials and methods

### Cell culture

Cell lines MCF10A (CRL-10317), MDA-MB-231 (HTB-26), MCF-7 (HTB-22), T47D (HTB-133), HCC1143 (CRL-2321) and MDA-MB-468 (HTB-132) were obtained from the American Type Culture Collection (LGC Standards GmbH, Wesel, Germany). MDA-MB-231 cells were maintained in Leibovitz’s L-15 medium (10% FBS, 1% L-glutamine, 1% nonessential amino acids (NEAA), 50 units/ml penicillin and 50 *μ*g/ml streptomycin sulfate (all from Invitrogen AG, Carlsbad, CA, USA)). MCF-7 were cultured in MEM (10% FBS, 1% NEAA, 0.01 mg/ml bovine insulin (Sigma-Aldrich, St. Louis, MO, USA), 50 units/ml penicillin and 50 *μ*g/ml streptomycin sulfate). MCF10A cells were cultured in DMEM F12 medium (5% horse serum (Thermo Fisher Scientific, Waltham, MA USA), 20 ng/ml EGF (BD Biosciences, Franklin Lakes, NJ, USA), 0.5 *μ*g/ml Hydrocortisone (Sigma-Aldrich), 100 ng/ml cholera toxin (Sigma-Aldrich), 0.01 mg/ml bovine insulin (Sigma-Aldrich), 50 units/ml penicillin and 50 *μ*g/ml streptomycin sulfate). T47D were cultured in RPMI medium (10% FBS, 1% NEAA, 50 units/ml penicillin and 50 *μ*g/ml streptomycin sulfate) and MDA-MB-468 and HCC1143 cells were cultured in RPMI medium (10% FBS, 50 units/ml penicillin and 50 *μ*g/ml streptomycin sulfate). PBMCs from healthy donors were isolated by Ficoll separation (LSM 1077 lymphocyte separation medium; PAA Laboratories, Pasching, Austria). NK cells were purified by negative selection (Human NK cell isolation kit; Miltenyi Biotec, Bergisch-Gladbach, Germany) with a purity of CD3-CD56+ NK cells >95%. NK cells were incubated in SCGM medium (CellGenix, Freiburg, Germany) containing 10% human serum (PAA Laboratories), 1% penicillin and 1% streptomycin (Invitrogen) with 200 IU/ml IL-2 (National Institutes of Health, Bethesda, MD, USA) overnight for the CD107a assay and for 2 days for ^51^Cr release assays. All cells lines were authenticated by Multiplexion (Heidelberg, Germany) and negatively tested for mycoplasma contamination before and after completion of the study.

### Transfections and reagents

Transfections of siRNA, miRNAs, miRNA vectors, gene expression vectors and luciferase vectors were performed using Lipofectamine 2000 or Lipofectamine RNAiMax (both from Invitrogen) according to the manufacturer’s instructions. ON TARGETplus siRNAs targeting TRAIL-R2, CASP7, CASP8 and TP53 were from Dharmacon (Lafayette, CO, USA). For each gene, three to four individual siRNAs were pooled (listed in Supplementary Table S2). ON TARGETplus nontargeting siRNA pool (Dharmacon) was used as control. miRIDIAN miRNA mimics miR-519a-3p (5′-AAAGUGCAUCCUUUUAGAGUGU-3′), miRNA hairpin inhibitors and negative controls (miRNA control and inhibitor control) were obtained from Dharmacon. The siRNAs, miRNA mimics and miRNA inhibitors were used at a final concentration of 30 or 50 nM. pCMV-MIR vector MIR519A2 (MI0003182) as well as empty vector control were obtained from OriGene (Rockville, MD, USA) and stable MDA-MB-231 cells were generated using G418 (Thermo Fisher Scientific). MCF10A cells were stably transfected with MICA, ULBP2^75^ or EMPTY vector using pmx-pie retroviral vectors and amphotropic Phoenix packaging cells as previously described,^76^ and then selected in complete medium containing 1 *μ*g/ml puromycin. To analyze cell proliferation, cell viability and/or apoptosis, the following reagents were used: TRAIL (PeproTech, Rocky Hill, NJ, USA), mouse anti human anti-Fas antibody (APO-1-3) used as ‘FasL’, granzyme B, perforin (both from Enzo Life Sciences, Farmingdale, NY, USA), staurosporine (Roche, Basel, Switzerland) and paclitaxel (Sigma-Aldrich). Concentrations were used as indicated and control cells were treated with respective solvents.

### Luciferase reporter assays

To validate direct targeting of miR-519a-3p, 3′UTR of the putative target genes TRAIL-R2, caspase-7, caspase-8, ULBP2, MICA and CD155 were cloned in the psiCHECK2 vector (Promega, Fitchburg, WI, USA) as previously described.^26^ Vectors, containing the respective 3′UTRs, were co-transfected with mimic miRNAs in MCF-7 or MCF10A cells. At 48 h after transfection, *Renilla* and Firefly luciferase activities were determined using a luminometer (Tecan, Männedorf, Switzerland). Mutations within each of the predicted target sites of MICA, ULBP2, TRAIL-R2 and caspase-8 3′UTRs were generated by site-directed mutagenesis using QuikChange II Site-Directed Mutagenesis Kit (Agilent Technologies, Santa Clara, CA, USA) according to the manufacturer’s instructions (Supplementary Table S3).

### Antibodies, immunoblotting and flow cytometry

For western blotting, cells were lysed in ice-cold M-PER lysis buffer (Thermo Fisher Scientific) containing NaF, Na_3_VO_4_, protease inhibitor Complete Mini (Roche) and phosphatase inhibitor PhosSTOP (Roche). Protein concentrations were determined by BCA Protein Assay Reagent Kit (Thermo Fisher Scientific) and proteins were denatured with 4 × Roti Load (Carl Roth, Karlsruhe, Germany) at 95 °C for 5 min. Depending on the size, proteins were separated by 12 and 15% SDS-PAGE, blotted onto a PVDF membrane Immobilon-FL (Merck Millipore, Darmstadt, Germany) and exposed to primary antibodies. The following antibodies were used: purified mouse anti-caspase-7 (clone C7, 9494; CST, Danvers, MA, USA), caspase-8 (clone 1C12, 9746, CST) and *β*-actin (MP Biochemicals, Santa Ana, CA, USA) as a loading control for each gel. Blots were probed with IRDye680- or IRDye780-conjugated secondary antibodies (H+L) and bands were visualized using an Odyssey scanner (LI-COR, Lincoln, NE, USA). Primary antibodies were used in a 1 : 1000 dilution and secondary antibodies in a 1 : 10 000 dilution. For flow cytometry, the following antibodies were used: purified mouse anti-MICA (clone 159227; R&D Systems, Wiesbaden-Nordenstadt, Germany), MICB (clone 236511; R&D Systems), ULBP1 (clone 170818, R&D Systems), ULBP2 (clone 165903, R&D Systems), ULBP3 (clone 166510, R&D Systems), ULBP4 (clone 709116, R&D Systems), mouse anti-CD155 (clone PV.404; Beckman Coulter, Brea, CA, USA), mouse anti-TRAIL-R1 (clone HS101), mouse anti-TRAIL-R2 (clone HS201), mouse anti-Fas antibodies (clone Apo-1-3, all three from Enzo Life Sciences) and IgG Isotype control (Sigma-Aldrich). Secondary FITC Goat anti-Mouse IgG/IgM or APC Goat anti-Mouse antibodies (both from BD Biosciences) were used. Cells were analyzed by flow cytometry (FACSCalibur, BD Biosciences) and FlowJo 9.3.2 software (Treestar, Ashland, OR, USA).

### RNA isolation and quantitative real-time PCR

Total RNA was isolated from cells using RNeasy Mini kit (Qiagen, Venlo, The Netherlands) according to the manufacturer’s instructions. For mRNA, cDNA synthesis was carried out with the Revert Aid H Minus First Strand cDNA Synthesis Kit (Fermentas, Waltham, MA, USA). The quantitative RT-PCR (qRT-PCR) reactions for target genes were performed using ABI Prism 7900HT Sequence Detection System (Applied Biosystems, Foster City, CA, USA), using probes from the Universal Probe Library (Roche) (listed in Supplementary Table S4). The housekeeping genes ACTB and TFRC were used for normalization of mRNA analysis. MicroRNA was isolated from cells using miRNeasy Mini kit (Qiagen) according to the manufacturer’s instructions. For miRNAs, the TaqMan microRNA reverse transcription kit and TaqMan gene-specific microRNA assays (Applied Biosystems) were used. For the qRT-PCRs, RNU44 and RNU48 were used as housekeeping controls. Data were acquired using a HT-7900 TaqMan instrument (Applied Biosystems) and analyzed with the ΔΔCT algorithm.

### Viability assays

RTCA (real-time cell analyzer) viability assay measures the effect of any perturbations in a label-free real-time setting. Electrical impedance (cell index (CI)) increases when cells adhere to the electronic sensors on bottom of the well. The increase in the impedance correlates with increasing cell numbers and cell adhesion. For RTCA experiments, transfections were performed as described above. Cells were seeded in RTCA E-plate 16 (Omni Life Science, Bremen, Germany) in full growth medium. After initial growth (18–36 h), cells were treated with 250 ng/ml TRAIL, 10 *μ*g/ml FasL or medium and additional cell growth was measured. For cell viability assay on MDA-MB-231, cells were treated with different concentrations of paclitaxel (Taxol) as indicated in the figure legends for 72 h and measured using the CellTiter-Glo Luminescent Cell Viability Assay kit (Promega). The assay was performed using the manufacturer’s protocol.

### Apoptosis assays

Quantification of DNA fragmentation was performed by FACS analysis of propidium iodide-stained nuclei as previously described,^77^ using a FACSCalibur flow cytometer (BD Biosciences) and the FlowJo software system. Apoptosis detection using Annexin V and 7-AAD was performed as described by the manufacturer’s protocol (BioLegend, San Diego, CA, USA). Caspase-3/7 activity was measured using the caspase-3/7 glo kit (Promega). The assay was performed using the manufacturer’s protocol. For analyzing mitochondrial membrane potential, cells were harvested using 0.25% trypsin, washed once with PBS and stained with 50 nM 1,1'dihexadecyl-3,3,3',3'-tetramethylin-docarbocyanine perchlorate (DiIC_1_(5)) according to the manufacturer’s protocol (Thermo Fisher Scientific) and analyzed by flow cytometry (FACS Calibur). To induce death receptor-mediated apoptosis, we used the monoclonal antibody anti-APO-1 IgG3 *κ* for CD95-mediated apoptosis and TRAIL at concentrations as indicated in the figure legends. To block caspase-mediated apoptosis, 25 *μ*M of broad-spectrum caspase inhibitor ZVAD-fmk (R&D Systems) was applied 30 min prior and during TRAIL and anti-Fas antibody treatment.

### ^51^Cr release assay

Target cells (0.5–1 × 10^6^ per 100 *μ*l) were labeled with 100 *μ*l of Na-chromate (^51^Cr, ∼3.7 MBq) (Perkin Elmer, Waltham, MA, USA) for 90 min at 37 °C. After three times washing, 2.5 × 10^4^ target cells were added to purified IL-2-activated NK cells at increasing effector/target (E/T) ratios in triplicate in 200 *μ*l. When indicated, NK cells were incubated with 10 *μ*g/ml of anti-NKG2D (1D11) or isotype control for 20 min before the addition of target cells. After 4 h, 50 *μ*l of cell-free supernatants were harvested, transferred to LumaPlate-96 (Perkin Elmer) and air-dried overnight. Release of ^51^Cr was measured with a TopCount NXT *γ*-counter (Perkin Elmer). Spontaneous and total ^51^Cr release was obtained by incubating targets cells in medium and 5% Triton X-100 (Sigma-Aldrich), respectively. Specific release (%)=(mean c.p.m. (sample)–mean c.p.m. (min))/(mean c.p.m. (max)−mean c.p.m. (min)), *n*=3. Assay medium was RPMI-1640 (Sigma-Aldrich), supplemented with 10% FCS and 100 IU/ml penicillin and 100 *μ*g/ml streptomycin (Sigma-Aldrich).

### CD107a degranulation assay

1 × 10^5^ NK cells were added to tumor cell lines at an E/T ratio of 1 : 1 in 200 *μ*l, spun down at 300 r.p.m. for 2 min and incubated in the presence of FITC-conjugated anti-CD107a antibody or isotype control antibody (BioLegend). After 30 min, 1 *μ*g of GolgiStop (BD Biosciences) was added for an additional period of 3.5 h. As a positive control for NK cell degranulation, NK cells were incubated with 50 ng/ml PMA and 1 mM ionomycin (Sigma-Aldrich). When indicated, NK cells were incubated with 10 *μ*g/ml of anti-NKG2D (1D11) or isotype control for 20 min before the addition to target cells. Cell suspensions were washed, stained for CD45-Pacific blue, CD3-PE and CD56-PE Cy7 (all from BioLegend) on ice in FACS buffer for 30 min, washed and briefly incubated with a 1 : 40 dilution of 7-AAD (BioLegend). Cells were measured on a FACS Canto II (BD Biosciences) and analyzed using FlowJo 9.3.2 software (Treestar).

### Analysis of patient data and statistical analysis

To test whether the expression of miR-519a-3p, TRAIL-R2, CASP7 and CASP8 correlated with disease-free or relapse-free survival, the data sets GSE19783 (patients, *n*=101) and GSE22220 (patients, *n*=216) were downloaded from the NCBI GEO database (GEO Accession GSE19783 and GSE22220) and the meta-analysis data set from KM Plotter version 2016 (patients, *n*=3554)^78^ were used for human primary breast tumors. To test whether miR-519a-3p is differentially expressed in ER− and ER+ as well as wild-type and mutant *TP53* data sets GSE19783 (patients, *n*=101) and GSE22220 (patients, *n*=216) were used, respectively. Correlations and statistical analyses were carried out using R packages ‘survival’ and ‘boxplot’ to generate Kaplan–Meier curves and boxplots. KM Plotter was used as an online tool. Log-rank and Student’s *t*-tests were performed. All *P*-values were calculated by means of a two-sided *t*-test where *P*-values of <0.05 were considered as significant, unless otherwise stated. Kaplan–Meier survival curves were carried out in GraphPad software (GraphPad software Inc., La Jolla, CA, USA). Enriched pathways predicted to be targeted by miR-519a-3p were identified using first the TargetScan (version 7.1) miRNA target prediction algorithm^79^ and then KEGG (Kyoto Encyclopedia of Genes and Genomes) analysis within the functional enrichment tool DAVID Bioinformatics Resources (version 6.7)^80^ (Supplementary Table S1).
