# Supplementary material for: MicroRNA-519a-3p mediates apoptosis resistance in breast cancer cells and their escape from recognition by natural killer cells
Source: Cell Death Dis. 2017 Aug 3;8(8):e2973–. doi: 10.1038/cddis.2017.364 (PMC5596553; doi:10.1038/cddis.2017.364)
Supplement: Supplementary Information [file cddis2017364x1.pdf]

Supplemental Information

MicroRNA-519a-3p mediates apoptosis resistance in breast cancer cells and their escape from recognition by Natural Killer cells

Christian Breunig, Jens Pahl, Moritz Küblbeck, Matthias Miller, Daniela Antonelli, Nese Erdem, Cornelia Wirth, Rainer Will, Alexander Bott, Adelheid Cerwenka, Stefan Wiemann

SUPPLEMENTARY TABLES

Table S1 KEGG pathway analysis for miR-519a-3p target genes. Predicted miR-519a-3p target genes from Target Scan were analysis within the functional enrichment tool DAVID Bioinformatics Resources.

.

Category	Term	Count	%	PValue
KEGG_PATHWAY	hsa05200:Pathways in cancer	30	0,246305419	0,030744118
KEGG_PATHWAY	hsa04010:MAPK signaling pathway	24	0,197044335	0,064588075
KEGG_PATHWAY	hsa04144:Endocytosis	23	0,188834154	0,002027671
KEGG_PATHWAY	hsa04510:Focal adhesion	19	0,155993432	0,071232232
KEGG_PATHWAY	hsa04360:Axon guidance	16	0,13136289	0,012929972
KEGG_PATHWAY	hsa04350:TGF-beta signaling pathway	15	0,123152709	8,01E-04
**KEGG_PATHWAY**	**hsa04650:Natural killer cell mediated cytotoxicity**	**15**	**0,123152709**	**0,034713218**
KEGG_PATHWAY	hsa04270:Vascular smooth muscle contraction	13	0,106732348	0,043171255
KEGG_PATHWAY	hsa05210:Colorectal cancer	12	0,098522167	0,013794187
KEGG_PATHWAY	hsa05215:Prostate cancer	12	0,098522167	0,020621859
KEGG_PATHWAY	hsa04114:Oocyte meiosis	12	0,098522167	0,076969828
KEGG_PATHWAY	hsa05218:Melanoma	11	0,090311987	0,011368043
KEGG_PATHWAY	hsa04012:ErbB signaling pathway	11	0,090311987	0,041339364
**KEGG_PATHWAY**	**hsa04210:Apoptosis**	**11**	**0,090311987**	**0,041339364**
KEGG_PATHWAY	hsa04540:Gap junction	11	0,090311987	0,047175431
KEGG_PATHWAY	hsa05214:Glioma	10	0,082101806	0,014630372
KEGG_PATHWAY	hsa04070:Phosphatidylinositol signaling system	10	0,082101806	0,03781618
KEGG_PATHWAY	hsa04662:B cell receptor signaling pathway	10	0,082101806	0,040735153
KEGG_PATHWAY	hsa04115:p53 signaling pathway	9	0,073891626	0,057403433
KEGG_PATHWAY	hsa04730:Long-term depression	9	0,073891626	0,061606669
KEGG_PATHWAY	hsa05212:Pancreatic cancer	9	0,073891626	0,075349504
KEGG_PATHWAY	hsa05220:Chronic myeloid leukemia	9	0,073891626	0,09080145
KEGG_PATHWAY	hsa04710:Circadian rhythm	4	0,032840722	0,042344015

Table S2 Sequences of siRNAs targeting CASP7, CASP8, TNFRSF10B, MICA and ULBP2 as well as non-targeting siRNA control.

Gene	siRNA no.	Sequence
CASP7	J-004407-06	GGGCAAAUGCAUCAUAAUA
	J-004407-07	GAUCAGGGCUGUAUUGAAG
	J-004407-09	CCAGACCGGUCCUCGUUUG
CASP8	J-003466-13	GGACAAAGUUUACCAAAUG
	J-003466-14	GCCCAAACUUCACAGCAUU
	J-003466-15	GAUAAUCAACGACUAUGAA
	J-003466-16	GUCAUGCUCUAUCAGAUUU
TNFRSF10B (TRAIL-R2)	J-004448-05	GCAAAUAUGGACAGGACUA
	J-004448-06	GCAAGUCUUUACUGUGGAA
	J-004448-07	CAAGGUCGGUGAUUGUACA
	J-004448-08	UCAUGUAUCUAGAAGGUAA
MICA	J-187896-05	CUUCAGAGUCAUUGGCAGA
	J-187896-06	CCAUGAACGUCAGGAAUUU
	J-187896-07	CUGCAAAAUGUUAGUAGAU
	J-187896-08	UGCAGGAACUACGGCGAUA
ULBP2	J-015898-09	GGUGCUACCUGAUGGAAUU
	J-015898-10	GCUGACUAAACAAGAUAUA
	J-015898-11	UGAGCACGGUCUUGAUCAA
	J-015898-12	CUUUAGAGUGACAGGUUAA
ON-TARGETplus Non-Targeting siRNA Pool	D-001810-10	UGGUUUACAUGUCGACUAA
		UGGUUUACAUGUUGUGUGA
		UGGUUUACAUGUUUUCUGA
		UGGUUUACAUGUUUUCCUA

Table S3 Sequences of PCR primers used for cloning and mutagenesis of TRAIL-R2, CASP8, MICA and ULBP2.

Name	Sequence (5'->3')
TRAILR2_fwd_Xho1	CTCGAGTCACATGACCGGTACTGGAAGA
TRAILR2_rev_Not1	GCGGCCGCGTGAAACCCCGTCTCTACTAAAA
TRAILR2_Mut1_fwd	CATCCTGTAACTTTTCACTGGTGATGGCATTATTTTTATAAGC
TRAILR2_Mut1_rev	GCTTATAAAAATAATGCCATCACCAGTGAAAAGTTACAGGATG
TRAILR2_Mut2_fwd	CTTTCTGGTCTTTGGTGATCCATCCTCTCCCAC
TRAILR2_Mut2_rev	GTGGGAGAGGATGGATCACCAAAGACCAGAAAG
TRAILR2_Mut3_fwd	GCGTTGTCCCCTGGTGATCTGGAAGGCACAG
TRAILR2_Mut3_rev	CTGTGCCTTCCAGATCACCAGGGGACAACGC
CASP8_fwd_Xho	CTCGAGTCTTGATCTCCTGACCTCGTG
CASP8_rev_Not1	GCGGCCGCCACTGCAGCCTTGACCTCTTG
CASP8_Mut_fwd	CTTTATTAATTGTTTTGGTGATTTTTATAAGAGCTAAAG
CASP8_Mut_rev	CTTTAGCTCTTATAAAAATCACCAAAACAATTAATAAAG
ULBP2_fwd_XhoI	CTCGAGTCCTGTGAGCACGGTCTTG
ULBP2_rev_NotI	GCGGCCGCTCAGGACACGGAAAGAATC
ULBP2_Mut_fwd	CTGATGGAATTCCTGGTGATAAAGTTCTGGCTGAC
ULBP2_Mut_rev	GTCAGCCAGAACTTTATCACCAGGAATTCCATCAG
MICA_fwd_XhoI	CTCGAGCCAGTTGGGACGAGTGACC
MICA_rev_NotI	GCGGCCGCTGCAGCCTCCAACAACAAT
MICA_Mut_fwd	GAAACAGAGAAAATAAAAGGTGATATTTATTGTTGTTGGAGG
MICA_Mut_rev	CCTCCAACAACAATAAATATCACCTTTTATTTTCTCTGTTTC

Table S4 Sequences and Universal Probe Library (UPL) probe numbers for genes quantified by TaqMan qRT-PCR.

Name	Sequence (5´- 3´)	UPL Probe
MICA_left	ggcatcttccctttttgcac	#24
MICA_right	ggacagcaccgtgaggttat	#24
ULBP2_left	ccgctaccaagatccttctg	#56
ULBP2_right	ggatgacggtgatgtcatagc	#56
CD155_left	caacgtcaccaatgcccta	#85
CD155_right	ctgagtgctcactgggaggt	#85
TNFRSF10A (TRAIL-R1)_left	gggtccacaagaccttcaagt	#18
TNFRSF10A (TRAIL-R1)_right	tgcagctgagctaggtacga	#18
TNFRSF10B (TRAIL-R2)_left	agacccttgtgctcgttgtc	#18
TNFRSF10B (TRAIL-R2)_right	ttgttgggtgatcagagcag	#18
CD95 (Fas)_left	gtggacccgctcagtacg	#60
CD95 (Fas)_right	tctagcaacagacgtaagaacca	#60
CASP7_left	ccgagacttttagtttcgcttt	#57
CASP7_right	cctgatcatctgccatcgt	#57
CASP8_left	taggggactcggagactgc	#2
CASP8_right	tttctgctgaagtccatcttttt	#2
ACTB_left	ccaaccgcgagaagatga	#64
ACTB_right	ccagaggcgtacagggatag	#64
TFRC_left	cccagttgctgtcctgatataga	#61
TFRC_right	ttgagaaaacaatgcaaaatgtg	#61

SUPPLEMENTARY FIGURES

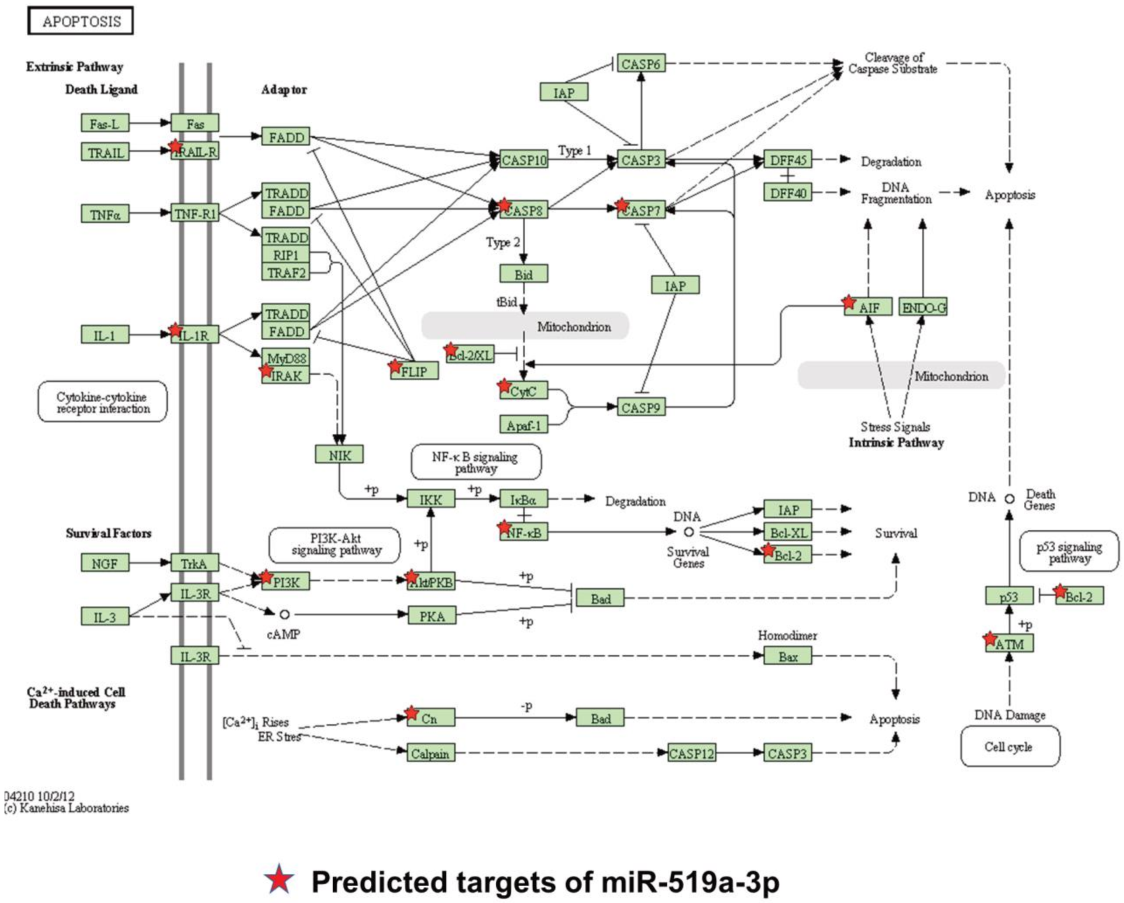

Figure S1 KEGG pathway analysis for apoptosis. MiR-519a-3p predicted targets were analyzed with the bioinformatical tool DAVID and the enriched KEGG pathway "Apoptosis" is shown. Predicted targets of miR-519a-3p are marked with the red star.

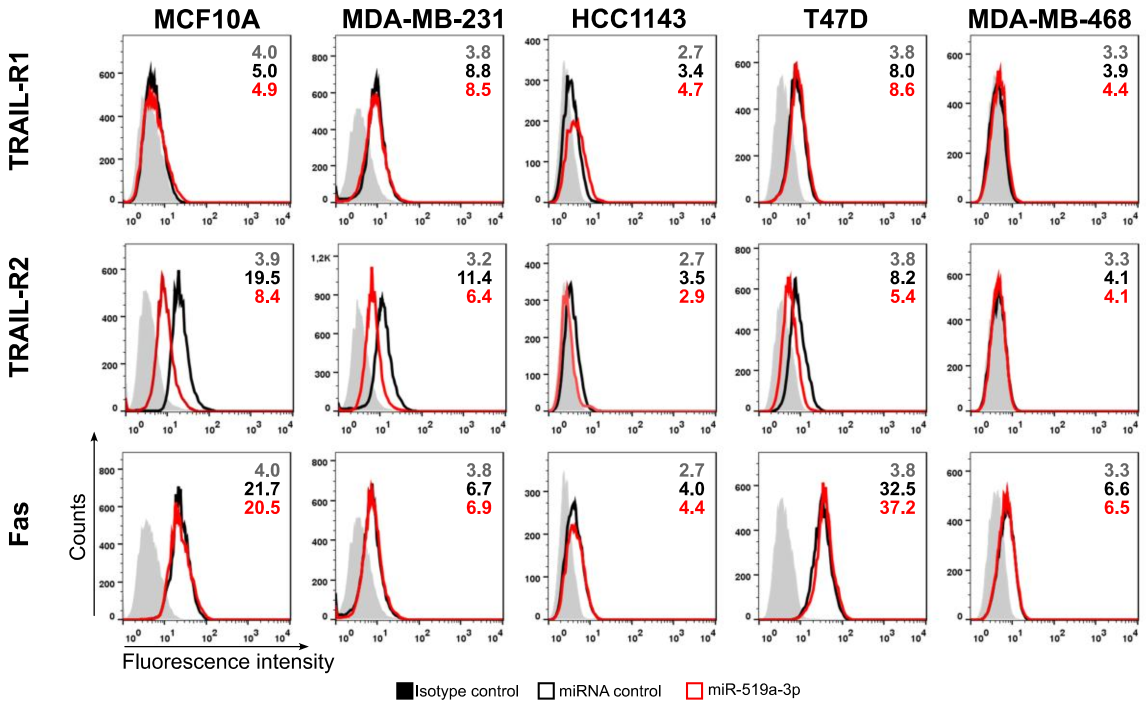

Figure S2 TRAIL-R2 is downregulated by miR-519a-3p in a panel of breast cancer cell lines. Flow cytometry analysis of TRAIL-R1, TRAIL-R2 and Fas after miR-519a-3p overexpression in breast cancer cells. Cells were transfected with miRNA control or miR-519a-3p for 48h and cells were stained for TRAIL-R1, TRAIL-R2 and Fas. MDA-MB-468 did not express TRAIL-R2 on the cell surface. Median fluorescence intensities for isotype control (grey), miRNA control (black) and miR-519a-3p (red) are depicted in each histogram. Shown is one representative FACS plot of at least three experimental repeats.

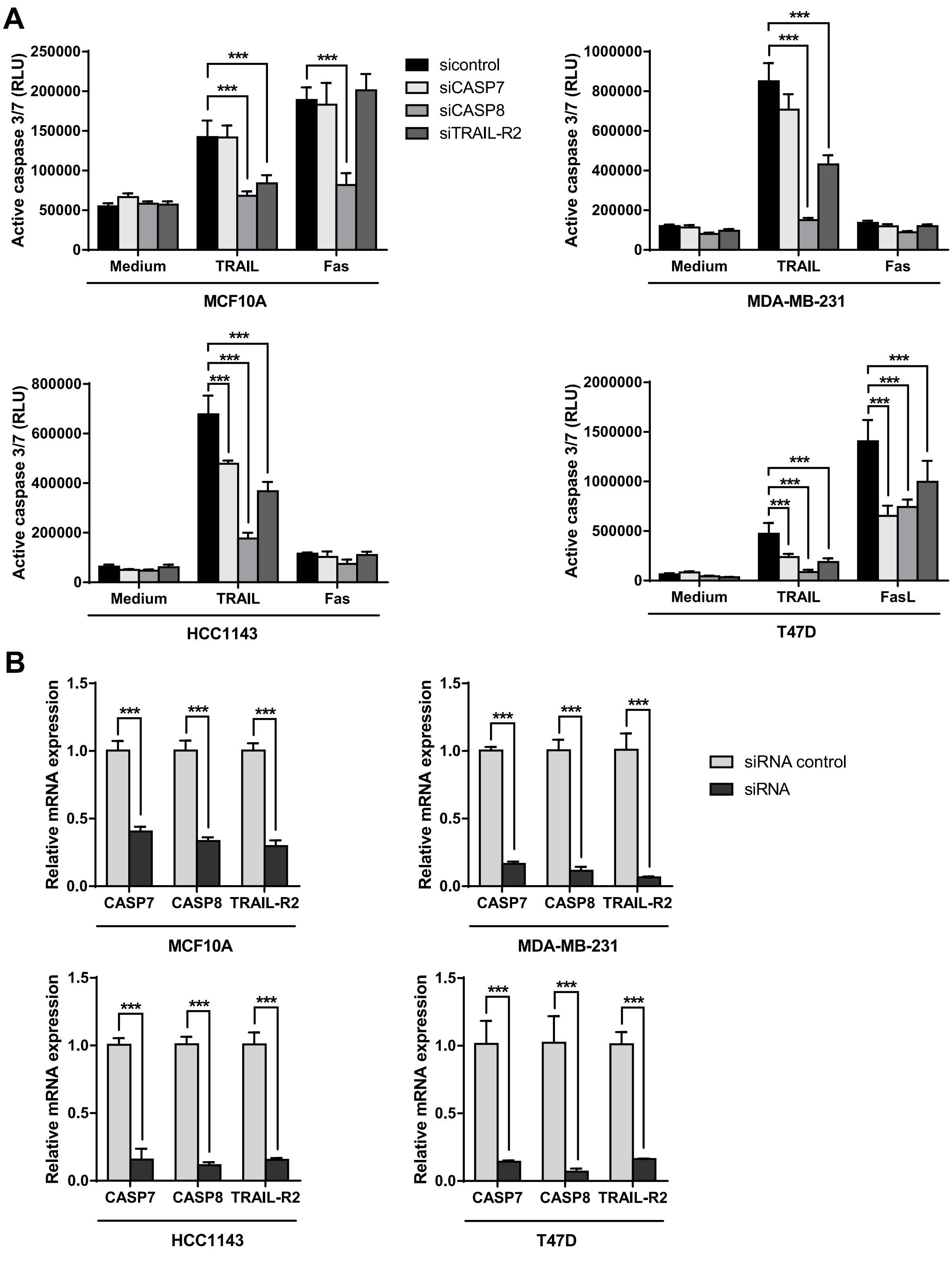
A
Active caspase 3/7 (RLU)
250000
200000
150000
100000
50000
0
***
Medium
TRAIL
Fas
MCF10A
sicontrol
siCASP7
siCASP8
siTRAIL-R2
1000000
800000
600000
400000
200000
MDA-MB-231
800000
600000
400000
HCC1143
2000000
1500000
1000000
500000
FasL
T47D
B
Relative mRNA expression
1.5
1.0
0.5
0.0
CASP7
CASP8
TRAIL-R2
siRNA control
siRNA

Figure S3 Effect of TRAIL-R2, caspase-7 and -8 on TRAIL- and FasL-induced apoptosis. (**A**) TRAIL-induced apoptosis was reduced by silencing TRAIL-R2 and caspase-8 in MCF10A, MDA-MB-231, HCC1143 and T47D cells. FasL-induced apoptosis was reduced by silencing caspase-8 in MCF10A cells and by caspase-7 and -8 in T47D cells. Cells were transfected with siTRAILR2, siCASP7, siCASP8 and siRNA control for 48h and then treated with 60 ng/ml TRAIL and 5 µg/ml FasL for MCF10A and 250ng/ml TRAIL and 20 µg/ml FasL for MDA-MB-231, HCC143 and T47D cells for 6h and active caspase 3/7 was measured (n=5). (**B**) qRT-PCR reveals downregulation of CASP7, CASP8 and TRAIL-R2 by siCASP7, siCASP8 and siTRAIL-R2, respectively, in MCF10A, MDA-MB-231, HCC1143 and T47D cells. Cells were transfected with siRNA control, siCASP7, siCASP8 or siTRAIL-R2 for 48h, mRNA was isolated and gene expression of *CASP7*, *CASP8* and *TNFRSF10B* (TRAIL-R2) was analyzed (n=3). Data are expressed as mean + SD; ***$p < 0.001$. All p values are based on analysis siRNA control versus siCASP7, siCASP8, siTRAIL-R2.

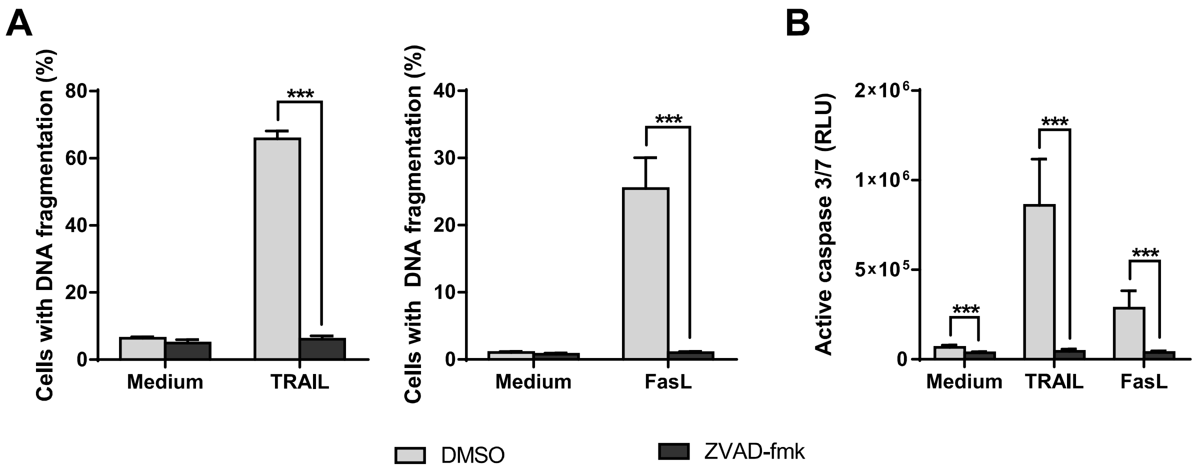

Figure S4 MiR-519a-3p induced apoptosis resistance is caspase dependent. (**A**) The pan caspase inhibitor ZVAD-fmk reduced 60 ng/ml TRAIL and 5µg/ml FasL-induced DNA fragmentation in MCF10A cells (n=3). (**B**) ZVAD-fmk reduced TRAIL and FasL-induced caspase 3/7 activity in MCF10A cells (n=8). Data are expressed as mean + SD; ***p < 0.001. All p values are based on analysis DMSO versus ZVAD-fmk.

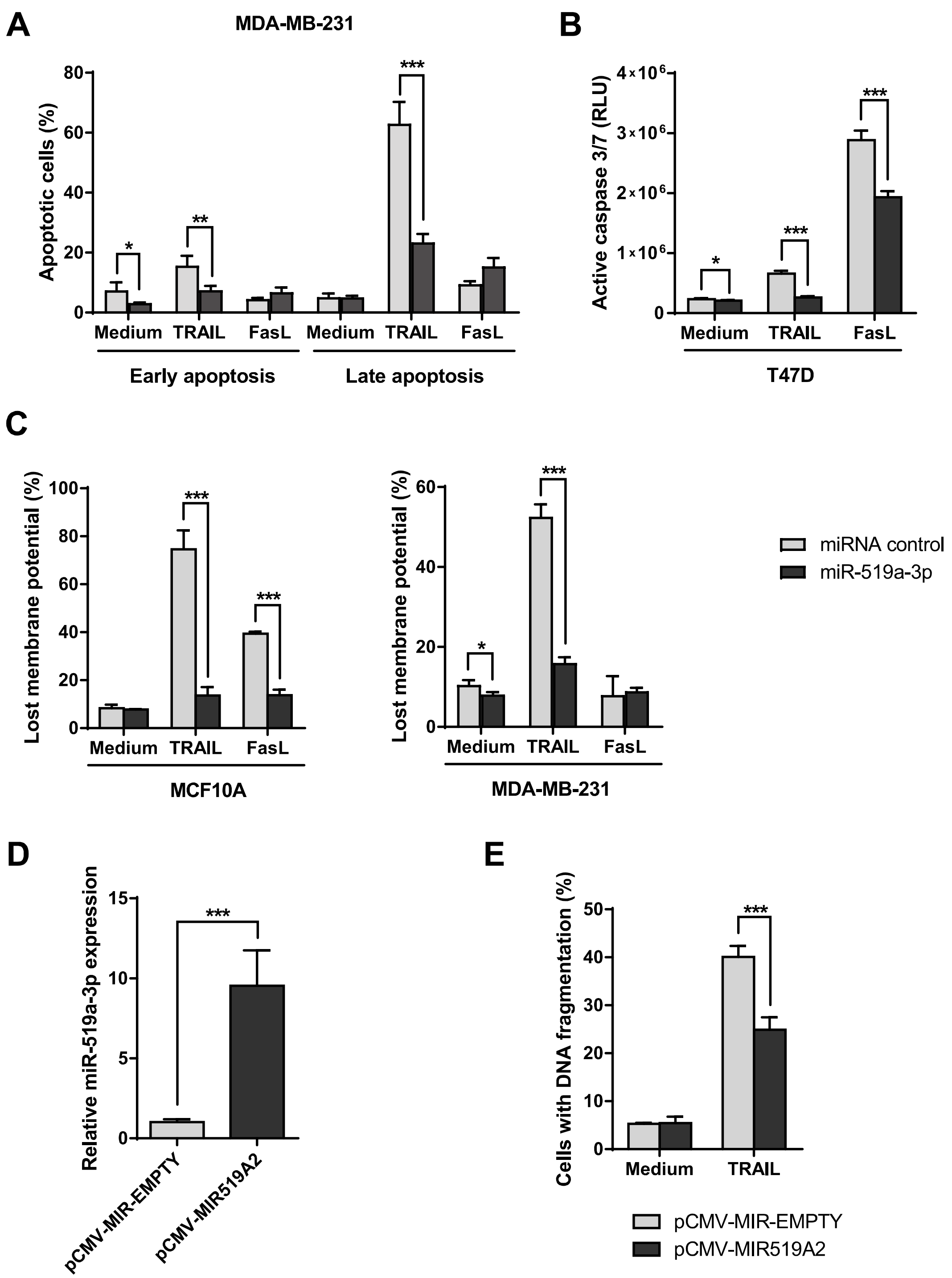
A
MDA-MB-231
Apoptotic cells (%)
Medium
TRAIL
FasL
Early apoptosis
Late apoptosis
B
Active caspase 3/7 (RLU)
T47D
C
Lost membrane potential (%)
MCF10A
MDA-MB-231
miRNA control
miR-519a-3p
D
Relative miR-519a-3p expression
pCMV-MIR-EMPTY
pCMV-MIR519A2
E
Cells with DNA fragmentation (%)
pCMV-MIR-EMPTY
pCMV-MIR519A2

Figure S5 Elevated expression of miR-519a-3p induces resistance towards apoptosis induction. (**A**) TRAIL and FasL-induced apoptosis was reduced by overexpression of miR-519a-3p in MDA-MB-231 cells using Annexin V and 7AAD. MDA-MB-231 cells were transfected with miR-519a-3p or miRNA control for 48h and then treated with 250 ng/ml TRAIL, 15 µg/ml FasL or medium control for additional 24h. Shown is the statistical analysis of early (Annexin V positive and 7AAD negative) and late (Annexin V positive and 7AAD positive) apoptosis (n=3). (**B**) Activation of caspase 3/7 activity in T47D cells by TRAIL and FasL was partly inhibited by miR-519a-3p (n=8). (**C**) MiR-519a-3p reduced TRAIL and FasL-induced loss of mitochondrial membrane potential in MCF10A and MDA-MB-231 cells (n=3). (**D**) MDA-MB-231 cells were generated to stably overexpress miR-519a-3p using a pCMV vector containing miR-519A2 gene. As a control the empty pCMV vector was used (n=5). (**E**) MDA-MB-231 cells stably overexpressing miR-519a-3p are more resistant towards TRAIL (250 ng/ml) treatment than control MDA-MB-231 cells (n=4). Data are expressed as mean + SD; *$p<0.05$, **$p <0.01$, ***$p < 0.001$. All p values are based on analysis miRNA control versus miR-519a-3p or empty vector versus MIR-519A2 vector.

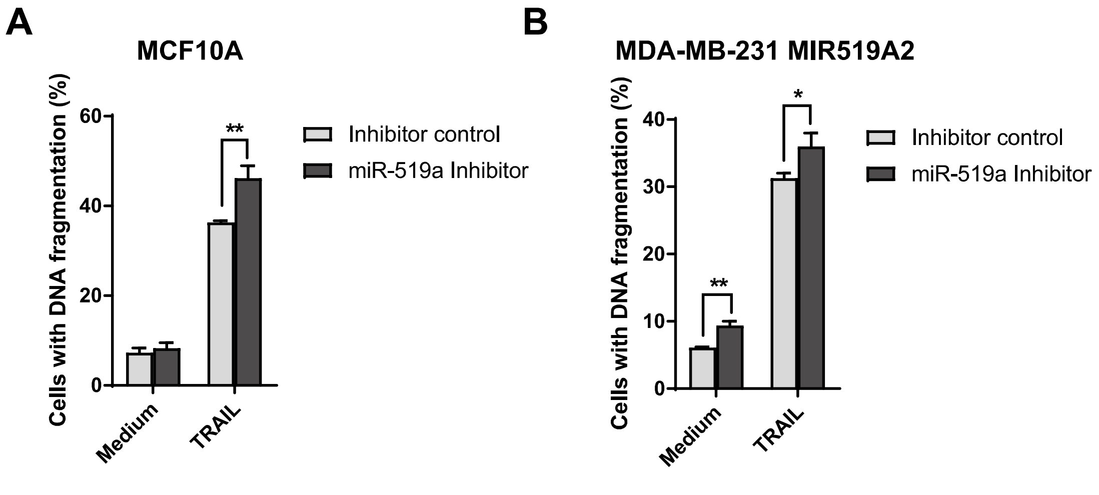

Figure S6 Inhibition of miR-519a-3p increased sensitivity towards apoptosis induction. (**A**) TRAIL (60 ng/ml)-induced DNA fragmentation was increased by miR-519a-3p inhibitor in MCF10A cells (n=6). (**B**) Inhibiting miR-519a-3p expression in MDA-MB-231 cells stably overexpressing miR-519a-3p increased apoptosis induction after 250 ng/ml TRAIL treatment (n=3). Data are expressed as mean + SD; *p<0.05, **p <0.01. All p values are based on analysis miRNA inhibitor versus miR-519a-3p inhibitor.

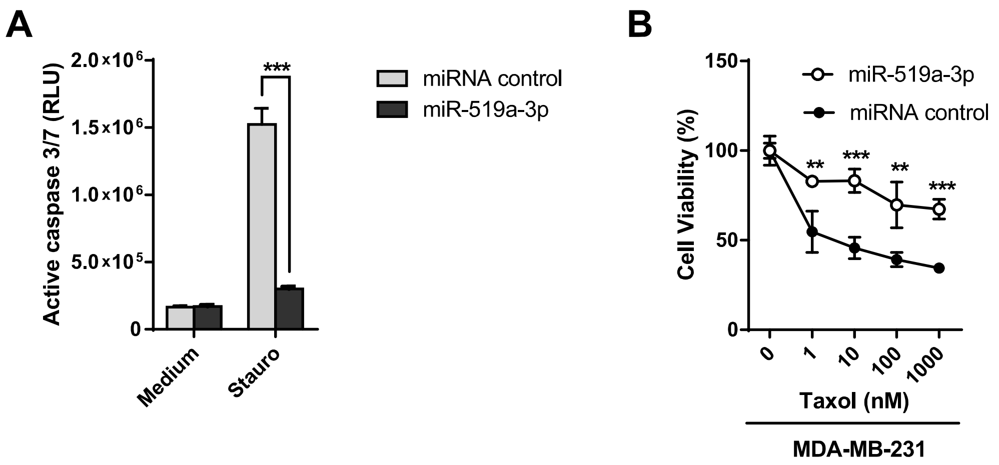

Figure S7 Elevated expression of miR-519a-3p induces resistance towards staurosporine and Taxol. (**A**) MCF10A cells were treated for 6h with 100 nM staurosporine induced activation of caspase 3/7 in miRNA control transfected, but not in miR-519a-3p transfected MCF10A cells (n=8). (**B**) Taxol reduced cell viability in a concentration dependent manner after 72h. Overexpression of miR-519a-3p induced resistance towards Taxol treatment in MCF10A cells (n=3). Data are expressed as mean + SD; **p <0.01, ***p < 0.001. All p values are based on analysis miRNA control versus miR-519a-3p.

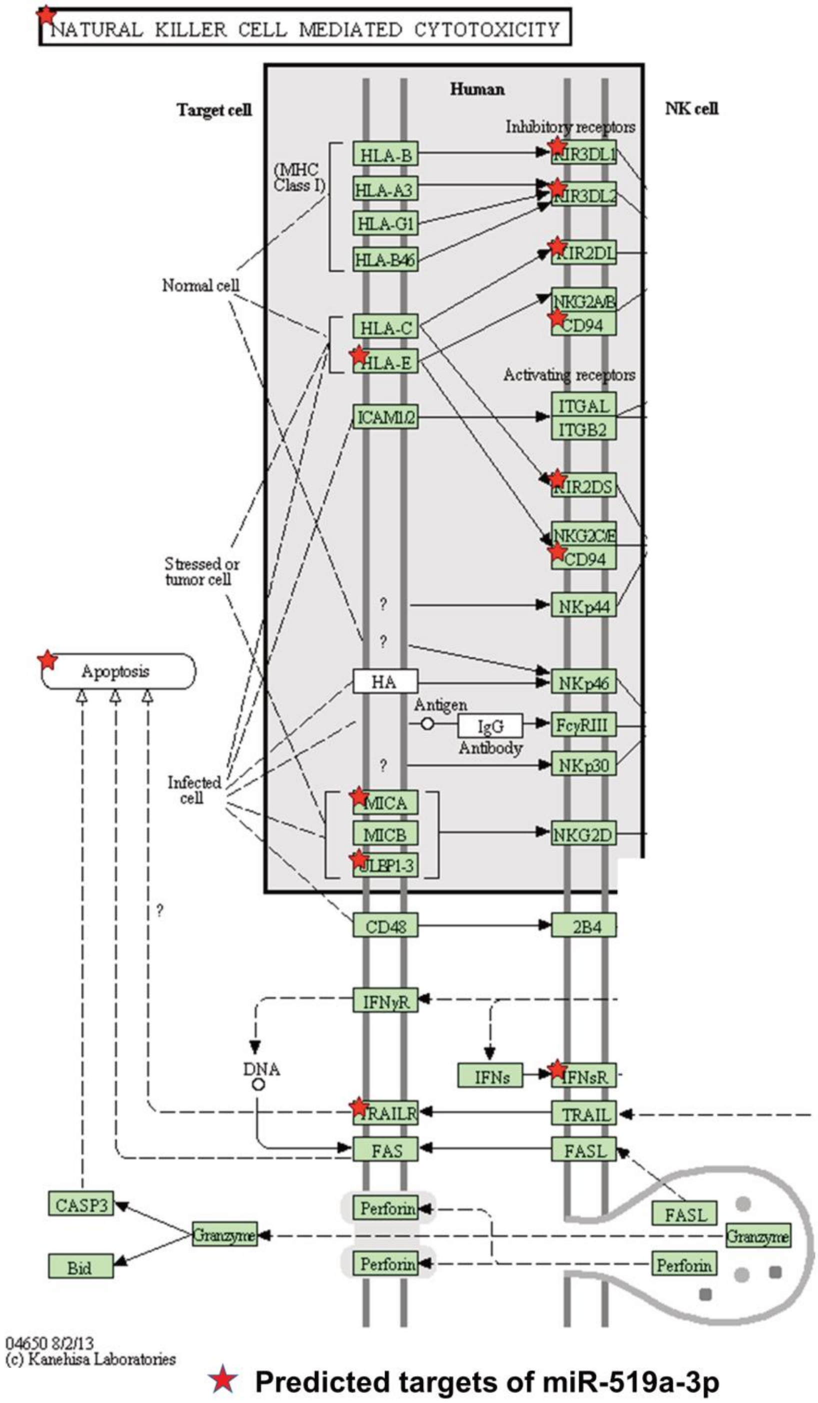

Figure S8 KEGG pathway analysis for NK cell-mediated cytotoxicity. MiR-519a-3p predicted targets were analyzed with the bioinformatical tool DAVID and the enriched KEGG pathway "Natural Killer cell mediated cytotoxicity" is shown. Predicted targets of miR-519a-3p are marked with the red star.

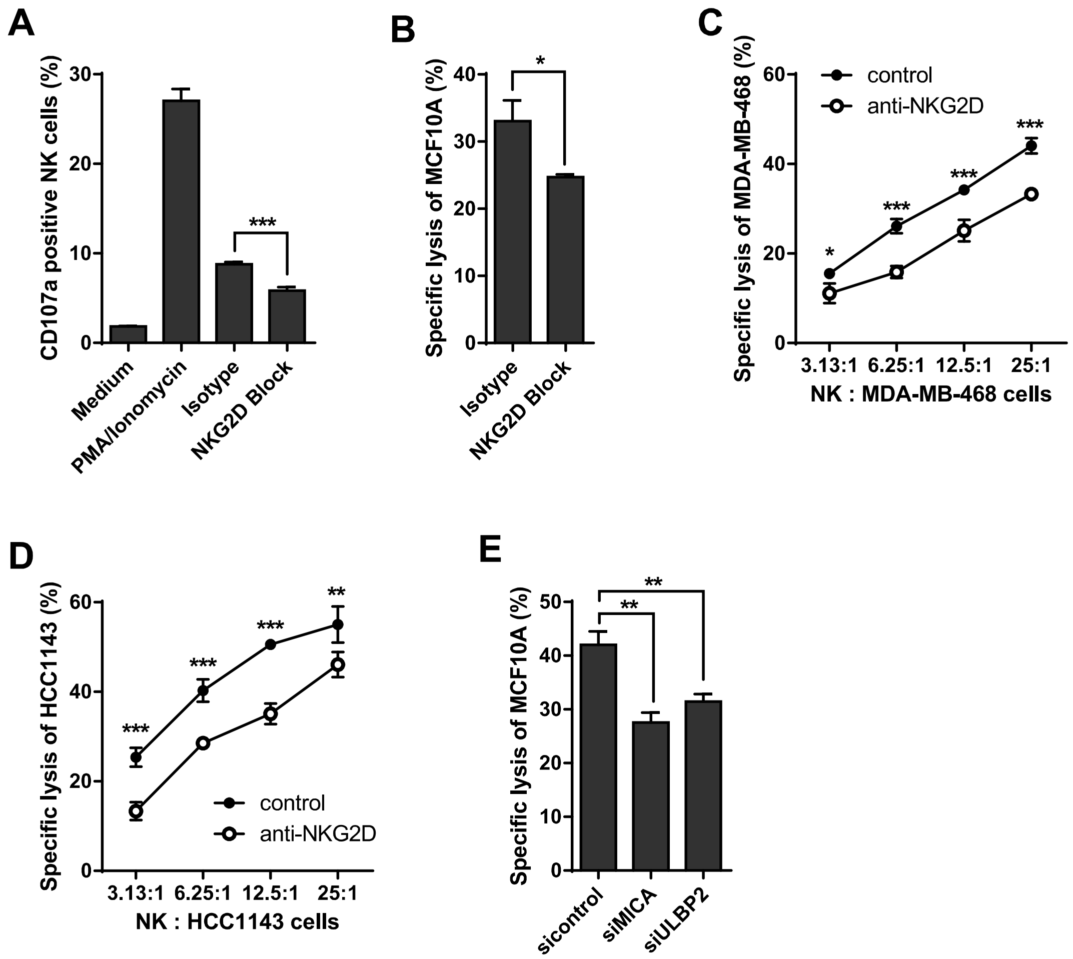

Figure S9 NKG2D and its ligand MICA and ULBP2 are important for NK cell-mediated cytotoxicity (**A**) Primary human NK cells were incubated with 10 µg/ml of anti-NKG2D or isotype control for 20 minutes prior to the addition of MCF10A cells in a 1:1 ratio and a CD107a assay was performed. Blocking NKG2D reduced NK cell activation (n=3). (**B-D**) NK cells were incubated with 10 µg/ml of anti-NKG2D or isotype control for 20 minutes prior to the addition of MCF10A, MDA-MB-468 and HCC1143 cells labeled with ^{51}Cr. Lysis of MCF10A, MDA-MB-468 and HCC1143 was measured in a 4h ^{51}Cr release assay. Blocking NKG2D reduced NK cytotoxicity (n=3) (**E**) MCF10A cells were transfected with siMICA, siULBP2 or siRNA control for 48h and then co-cultured with primary human NK cells. Lysis of MCF10Acells was measured in a 4h ^{51}Cr release assay. Reducing MICA and ULBP2 reduced NK cytotoxicity (n=3). Data are expressed as mean + SD; *p < 0.05, **p <0.01, ***p < 0.001. All p values are based on analysis NKG2D block versus Isotype control, siMICA or siULBP2 versus siRNA control.

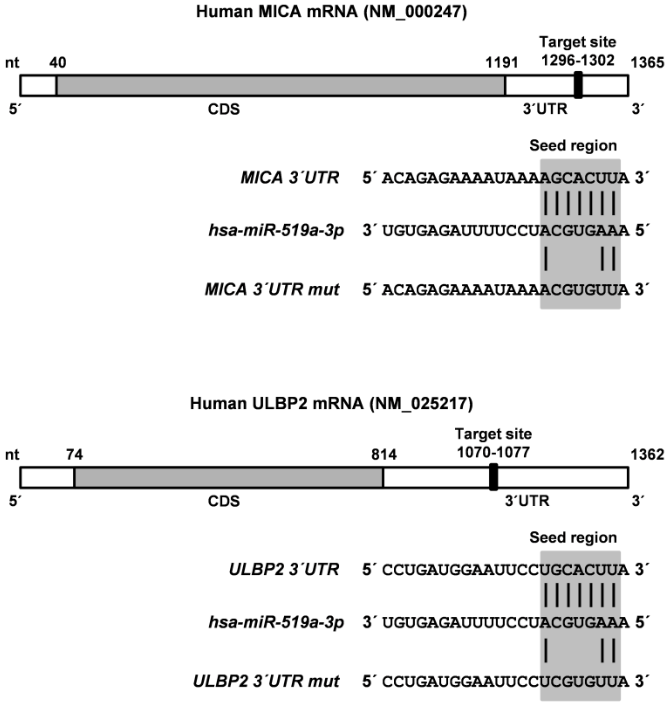

Figure S10 Schematic representation of the miR-519a-3p target sites within the 3´UTRs of MICA and ULBP2 mRNAs.

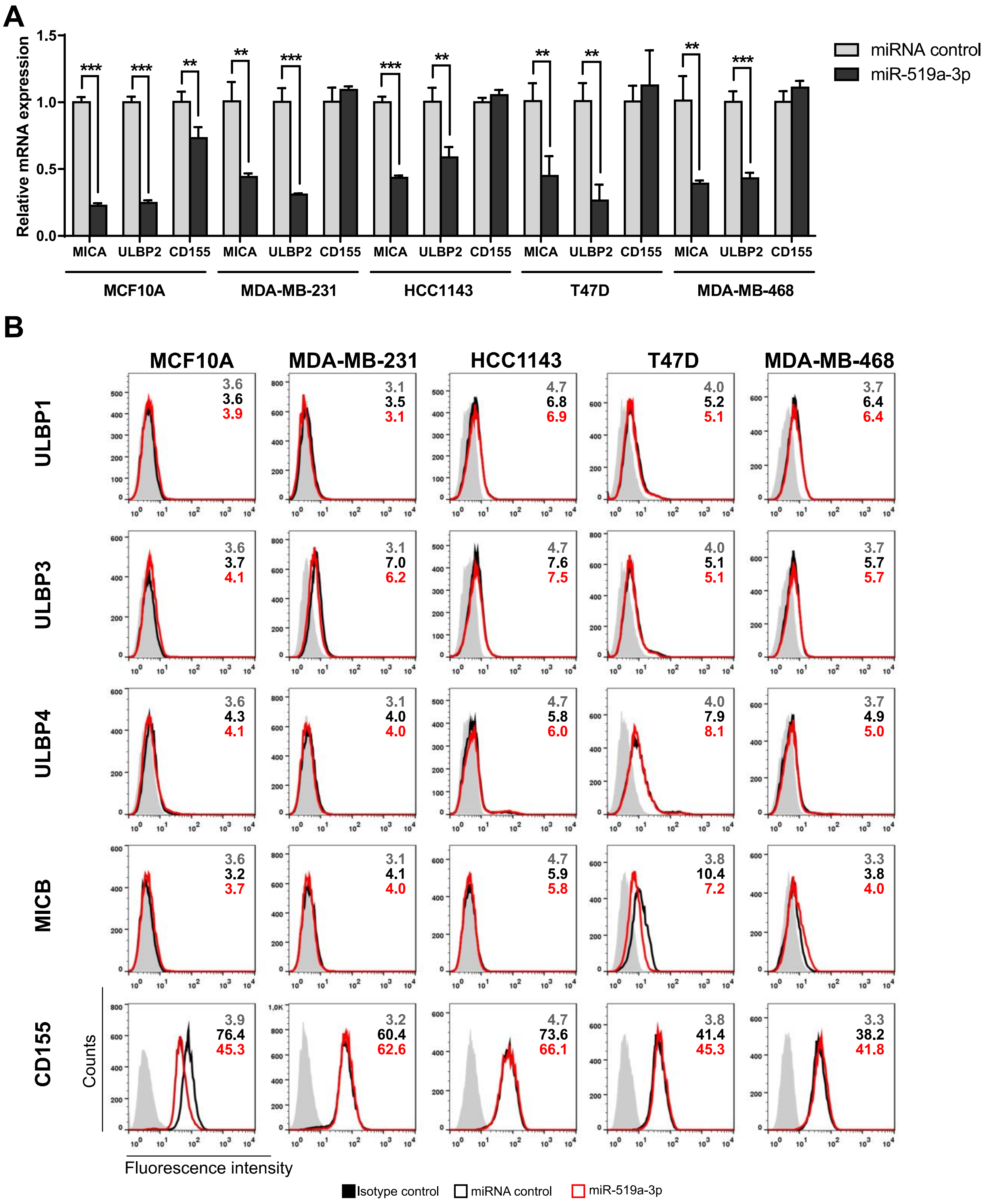
A
Relative mRNA expression
miRNA control
miR-519a-3p
MICA ULBP2 CD155 MICA ULBP2 CD155 MICA ULBP2 CD155 MICA ULBP2 CD155 MICA ULBP2 CD155
MCF10A
MDA-MB-231
HCC1143
T47D
MDA-MB-468
B
MCF10A
MDA-MB-231
HCC1143
T47D
MDA-MB-468
ULBP1
ULBP3
ULBP4
MICB
CD155
Counts
Fluorescence intensity
Isotype control
miRNA control
miR-519a-3p

Figure S11 ULBP2 and MICA are downregulated by miR-519a-3p in a panel of breast cancer cell lines. (**A**) qRT-PCR reveals downregulation of MICA and ULBP2 in MCF10A, MDA-MB-231, HCC1143, T47D and MDA-MB-468 cells. Breast cancer cells were transfected with miRNA control or miR-519a-3p for 48h, mRNA was isolated and gene expression of MICA, ULBP2 and CD155 was analyzed (n=3). (**B**) FACS analysis of ULBP1, ULBP3, ULBP4, MICB and CD155 of MCF10A, MDA-MB-231, HCC1143, T47D and MDA-MB-468 cells. Cells were transfected with miRNA control or miR-519a-3p for 48h and surface molecule expression was analyzed by FACSCalibur. Median fluorescence intensity for isotype control (grey), miRNA control (black) and miR-519a-3p (red) are depicted in each histogram. Shown is one representative FACS plot of at least three experimental repeats. Data are expressed as mean + SD; $^{**}p < 0.01$, $^{***}p < 0.001$. All p values are based on analysis miRNA control versus miR-519a-3p.

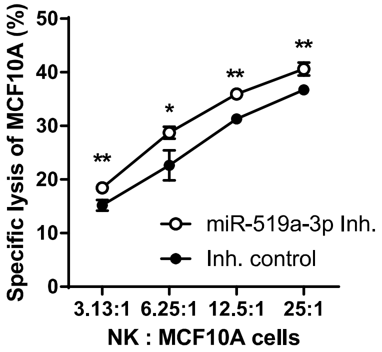

Figure S12 MiR-519a-3p influences NK cell-mediated cytotoxicity. MCF10A cells were transfected with miR-519a-3p inhibitor or miRNA inhibitor control for 48h and then co-cultured with primary human NK cells in different ratios, and chrome release assays were performed. Inhibition of miR-519a-3p increased killing of MCF10A by NK cells (n=3). Data are expressed as mean ± SD; *p < 0.05, **p <0.01. All p values are based on analysis miRNA inhibitor control versus miR-519a-3p inhibitor.

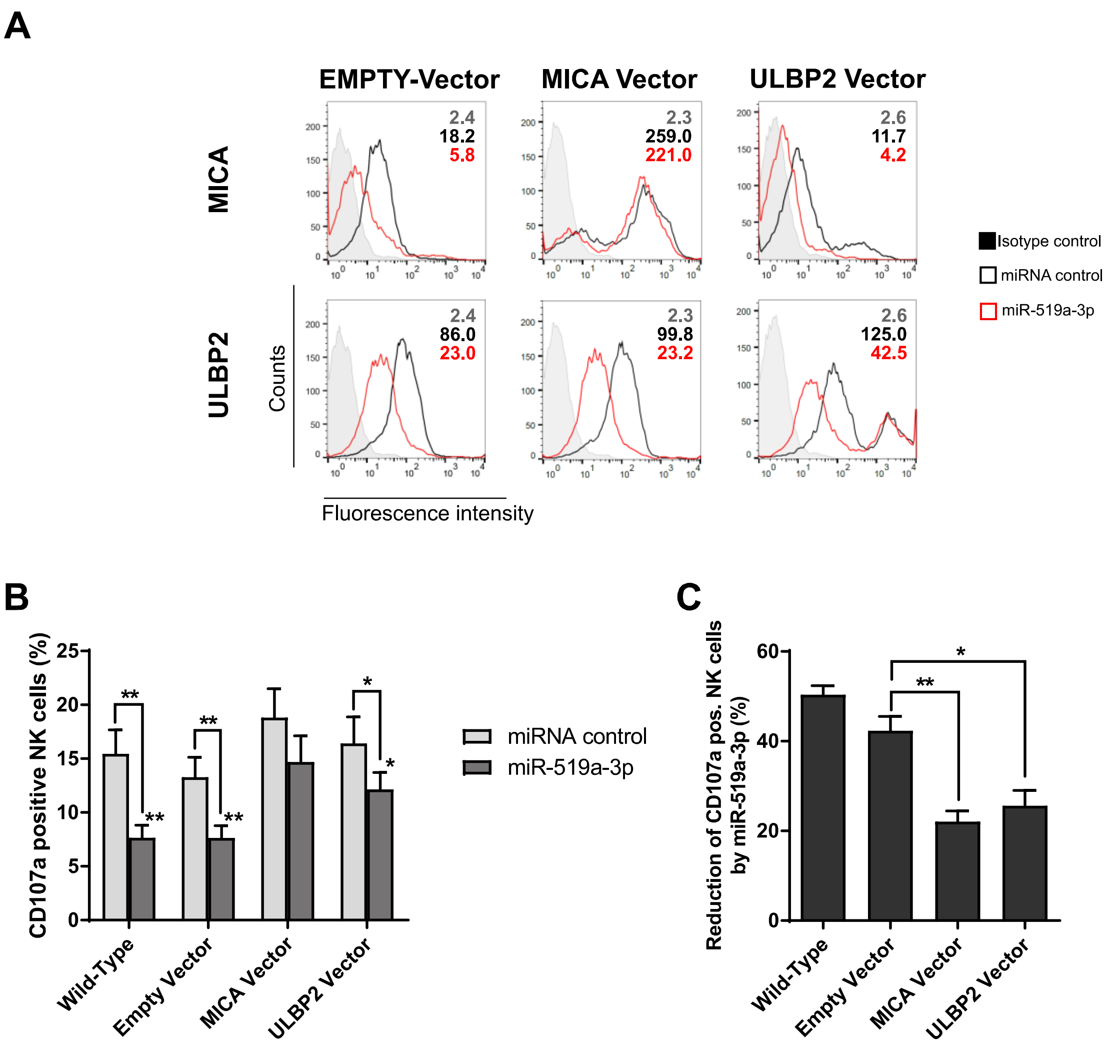

Figure S13 MiR-519a-3p influences NK cell-mediated cytotoxicity via MICA and ULBP2. (**A**) FACS analysis of MICA and ULBP2 of MCF10A stable transfected with MICA, ULBP2 or EMPTY vector and surface molecule expression was analyzed by FACSCalibur. Median fluorescence intensity for isotype control (grey), miRNA control (black) and miR-519a-3p (red) is depicted in each histogram. (**B**) Wild-type, Empty vector control, MICA stable overexpressing or ULBP2 stable overexpressing MCF10A cells were transfected with miR-519a-3p or miRNA control for 48h and then co-cultured with primary human NK cells in a 1:1 ratio and a CD107a assay was performed. MiR-519a-3p reduced NK cell activation and overexpression of MICA or ULBP2 reduced inhibitory effect of miR-519a-3p (n=4). (**C**) Percentage reduction of CD107a positive NK cells by miR-519a-3p compared to miRNA control in Wild-type, Empty vector control, MICA stable overexpressing or ULBP2 stable overexpressing MCF10A cells (n=4). Data are expressed as mean + SD; *p < 0.05, **p <0.01. All p values are based on analysis miRNA control versus miR-519a-3p or Empty vector versus MICA / ULBP2 vector.

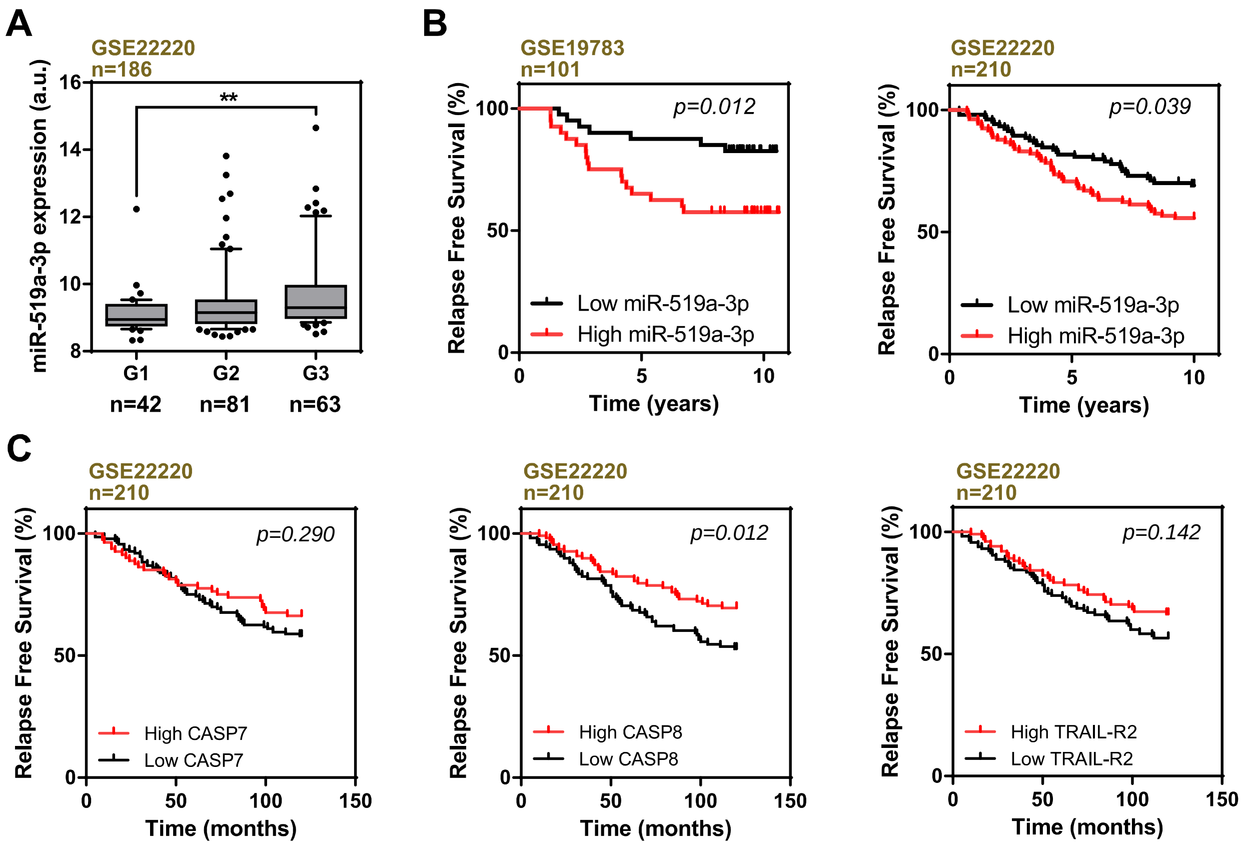

Figure S14 High miR-519a-3p and low *CASP7*, *CASP8* and *TNFRSF10B* (TRAIL-R2) levels correlate with survival outcome. (**A**) Analyzing breast cancer patient data set GSE22220 revealed that miR-519a-3p is higher expressed in histopathologic G3 breast cancer patients. (**B**) Higher miR-519a-3p expression correlates with poorer survival of breast cancer patients (GSE19783 and GSE22220). (**C**) Lower *CASP7*, *CASP8* and *TNFRSF10B* (TRAIL-R2) expressions (black curve) correlate with poorer survival of breast cancer patients (GSE22220).

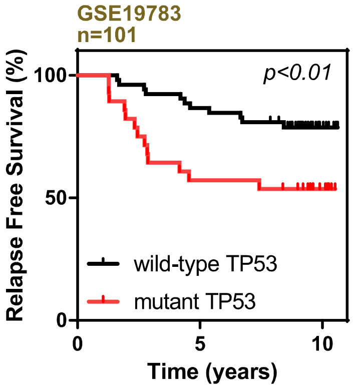

Figure S15 p53 regulates miR-519a-3p expression. Analyzing breast cancer patient data set GSE19873 revealed that mutant *TP53* correlates with poorer survival of breast cancer patients.
